# Mutations in α-synuclein, TDP-43 and tau prolong protein half-life through diminished degradation by lysosomal proteases

**DOI:** 10.1186/s13024-023-00621-8

**Published:** 2023-05-02

**Authors:** Paul J. Sampognaro, Shruti Arya, Giselle M. Knudsen, Emma L. Gunderson, Angelica Sandoval-Perez, Molly Hodul, Kathryn Bowles, Charles S. Craik, Matthew P. Jacobson, Aimee W. Kao

**Affiliations:** 1 Memory and Aging Center, Department of Neurology, University of California, San Francisco, CA USA; 2 Neuromuscular Division, Department of Neurology, University of California, San Francisco, CA USA; 3 Alaunus Biosciences, Inc., South San Francisco, CA USA; 4 Department of Pharmaceutical Chemistry, University of California, San Francisco, San Francisco, CA USA; 5 Department of Genetics and Genomic Sciences, Icahn School of Medicine at Mount Sinai, New York, USA; 6 UK Dementia Research Institute at the University of Edinburgh, Edinburgh Medical School, Edinburgh, UK

**Keywords:** Cathepsin, Protease, α-synuclein, TDP-43, Tau, Lysosome, Autophagy, Mutations, Neurodegeneration

## Abstract

**Background:**

Autosomal dominant mutations in α-synuclein, TDP-43 and tau are thought to predispose to neurodegeneration by enhancing protein aggregation. While a subset of α-synuclein, TDP-43 and tau mutations has been shown to increase the structural propensity of these proteins toward self-association, rates of aggregation are also highly dependent on protein steady state concentrations, which are in large part regulated by their rates of lysosomal degradation. Previous studies have shown that lysosomal proteases operate precisely and not indiscriminately, cleaving their substrates at very specific linear amino acid sequences. With this knowledge, we hypothesized that certain coding mutations in α-synuclein, TDP-43 and tau may lead to increased protein steady state concentrations and eventual aggregation by an alternative mechanism, that is, through disrupting lysosomal protease cleavage recognition motifs and subsequently conferring protease resistance to these proteins.

**Results:**

To test this possibility, we first generated comprehensive proteolysis maps containing all of the potential lysosomal protease cleavage sites for α-synuclein, TDP-43 and tau. In silico analyses of these maps indicated that certain mutations would diminish cathepsin cleavage, a prediction we confirmed utilizing in vitro protease assays. We then validated these findings in cell models and induced neurons, demonstrating that mutant forms of α-synuclein, TDP-43 and tau are degraded less efficiently than wild type despite being imported into lysosomes at similar rates.

**Conclusions:**

Together, this study provides evidence that pathogenic mutations in the N-terminal domain of α-synuclein (G51D, A53T), low complexity domain of TDP-43 (A315T, Q331K, M337V) and R1 and R2 domains of tau (K257T, N279K, S305N) directly impair their own lysosomal degradation, altering protein homeostasis and increasing cellular protein concentrations by extending the degradation half-lives of these proteins. These results also point to novel, shared, alternative mechanism by which different forms of neurodegeneration, including synucleinopathies, TDP-43 proteinopathies and tauopathies, may arise. Importantly, they also provide a roadmap for how the upregulation of particular lysosomal proteases could be targeted as potential therapeutics for human neurodegenerative disease.

**Supplementary Information:**

The online version contains supplementary material available at 10.1186/s13024-023-00621-8.

## Background

Despite symptomatic and anatomical differences, neurodegenerative diseases such as Parkinson’s disease (PD), amyotrophic lateral sclerosis (ALS) and frontotemporal dementia (FTD) are all characterized by the neuropathological finding of proteinaceous intraneuronal aggregates on which disease nosology is based and genetic cohorts have been identified [1]. The mechanistic basis for aggregate formation represents an area of active investigation, with recent cryo-EM studies revealing distinctive structural features of α-synuclein (α-syn), TDP-43 and tau inclusions [2–4]. Rates of protein self-association and aggregation depend sensitively on the steady-state levels of soluble protein present within a cell, which can increase as part of aberrant protein homeostasis [5, 6]. For α-syn, increased protein concentration alone serves as a causal factor for disease, given what the identification of cohorts with *SNCA* triplication and quadruplication leading to familial Parkinson's disease has shown [7, 8]. Mutations in the *SNCA, TARDBP* and *MAPT* genes that alter specific amino acids also increase the risk for neurodegenerative disease and are thought to do so by increasing the rate of protein self-association [9–11]. However, only a subset of α-syn, TDP-43 and tau mutations have been experimentally shown to increase aggregation potential while many other autosomal dominant disease mutations do not directly affect protein oligomerization [12–14] and yet, at least with TDP-43, nevertheless extend protein half-life [15, 16]. Thus, we hypothesized that certain remaining autosomal dominant mutations in α-syn, TDP-43 and tau may increase their own steady-state levels over time via an alternative mechanism.

Lysosomes play major roles in cellular protein homeostasis [17–20]. As the engines of autophagy, lysosomes have been increasingly recognized as key players in neurodegeneration, with mutations in lysosome-resident proteins including *GBA, PGRN* and *CTSD* implicated in PD, FTD and AD, respectively [21, 22]. Deficient lysosomal function has also been identified as an early pathogenic event in animal models of neurodegeneration [23]. Although α-syn, TDP-43 and tau are all subject to lysosomal clearance [24–26], past efforts have largely focused on the upstream mechanisms of autophagy and stopped at the lysosomal membrane, presuming that α-syn, TDP-43 and tau are each efficiently degraded by the lysosomal proteases thereafter. These proteases, most of which are known as cathepsins, can be classified as cysteine, serine, or aspartyl proteases based on the key catalytic amino acids present in their active sites. Cathepsins exhibit selectivity for their substrate clients through the recognition of specific linear sequences of amino acids. Modest pH alterations or single residue changes in the target sequence of substrate proteins can dramatically alter cleavage kinetics as well [27–30]. To date, however, the ability of cathepsins to cleave wild type (WT) versus mutant versions of these proteins has not been systematically explored.

In this study, we investigated the normal lysosomal clearance of α-syn, TDP-43 and tau and found that a subset of disease mutations extended protein half-life and increased steady-state protein levels. These insights arose following the generation and analysis of comprehensive maps of protease cleavage of these proteins. The maps revealed that areas dense with cleavage sites often overlapped with areas where disease mutations are concentrated. They also predicted mutations that were likely to interfere with protease cleavage. Systematic testing of a subset of these “disruptive” mutations validated these predictions in vitro as well as in cell and iPSC-derived neuronal (iNeuron) models. Disease mutations prolonged α-syn, TDP-43 and tau half-life and increase steady-state protein levels, even when similar rates of lysosomal import of mutant versus wild type protein were observed. Together, this study offers a novel, shared pathobiological mechanism by which genetic and potentially sporadic forms of age-related neurodegenerative disease may develop and implicates lysosomal proteases as therapeutically targetable contributors in neurodegeneration.

## Methods

### Recombinant proteases

Cathepsin E (CTSE) (R&D #1294-AS), cathepsin D (CTSD) (R&D #1014-AS), cathepsin G (CTSG) (Millipore #219,873), cathepsin A (CTSA) (R&D #1049-SE), cathepsin L (CSTL) (Millipore #219,402), cathepsin B (CTSB) (Millipore #219,364), cathepsin K (CTSK) (Millipore #219,461), (CTSS) cathepsin S (R&D #1183-CY), cathepsin V (CTSV) (R&D #1080-CY), asparagine endopeptidase (AEP) (R&D #2199-CY), cathepsin H (CTSH) (R&D #7516-CY-010), cathepsin C (CTSC) (R&D #1071-CY), cathepsin O (CTSO) (Abcam #ab267932), cathepsin F (CTSF) (Abcam #ab240858), and cathepsin X (CTSX) (R&D #934-CY).

### Antibodies

The following primary antibodies (commercial identifier, dilution) were used: 1) monoclonal mouse anti-GAPDH (Abcam #ab8245, 1:2500), 2) mouse anti-FLAG antibody (Sigma-Aldrich #F3040, 1:1000), 3) mouse anti-α-synuclein (Cell Signaling #2647S, 1:1000), 4) mouse anti-TDP-43 (Abcam #ab57105, 1:1000), 5) mouse anti-Tau (Santa Cruz #sc-1995, 1:1000), 6) anti-rabbit anti-Lamp1 antibody (Cell Signaling #9091, 1:1000), 7) rabbit anti-Cathepsin D (Cell Signaling # 2284, 1:1000), 8) mouse anti-Actin-C4 antibody (EMD Millipore #MAB1501, 1:1000), 9) rabbit anti-LC3b antibody (Sigma #L7543, 1:1000), and 10) rabbit anti-TFEB antibody (Proteintech #13,372, 1:1000).

### Plasmid constructs, point mutations, and stable lines

Novel doxycycline-inducible, FLAG-tagged, full-length WT Tau, TDP-43, and α-syn lentiviral plasmid constructs were first designed and synthesized (Epoch Life Sciences) using a puromycin resistant plasmid backbone, pTet-O-Ngn2-Puro (Addgene #52,047). Using these 3 WT plasmid constructs, individual constructs containing single pathogenic point mutations were also generated: Α-syn A30P, E46K, H50Q, G51D and A53T; TDP-43 G298S, A315T, A321G, Q331K and M337V; and tau K257T, N279K, P301S and S305N. With these constructs, lentivirus was made using the psPAX2 packing (Addgene #12,260) and pCMV-VSV-G envelope (Addgene #8454) plasmids. To generate stable lines, SH-SY5Y cells already containing the pLenti CMV rtTA3 Blast construct (Addgene #26,429) were infected and stable lines (17 in total) were generated using Puromycin 1 mg/mL selection.

### In vitro protease cleavage assays

For in vitro cleavage assays, 1 µg of recombinant full-length human α-syn (Abcam #ab51189), TDP-43 (R&D #AP-190) or 4N2R tau (rPeptide #T-1001–1) was incubated with or without 1 µM of each protease. Proteases requiring pre-activation were performed, as mentioned in Table S2. The following buffers were used as indicated: 100 mM sodium citrate pH 3.4, 50 mM sodium acetate pH 4.5 or 5.5 or 100 mM phosphate buffer saline (PBS) pH 7.4. Also, 1 mM EDTA and 2 mM DTT were used and each reaction was performed over 1 h at 37ºC. The assay was performed in a total volume of 19.5 µl. Protease activity was stopped by adding 7.5 µl of NuPAGE 4X LDS (Fisher Scientific #NP0007) and 3 µl of 10X reducing agent (i.e., 50 µM) (Fisher #NP0009). Samples were then immediately denatured for 10 min at 80ºC. All samples were run on precast NOVEX 4–12% Bis–Tris gels (Fisher #NP0321PK2) using MES buffer (Fisher #NP0002). The gel was then either fixed in 40% ethanol and 10% acetic acid for silver stain or transferred onto nitrocellulose membranes for Western blotting. Silver staining was performed according to the manufacturer’s instructions (Thermo Fisher #LC6070).

### Multiplexed substrate profiling by mass spectrometry (MSP-MS)

MSP-MS was performed as previously published by O’Donoghue et al. 2012 [31]. A substrate library was designed to cover the sequences for the three human proteins TDP-43, α-syn, and tau. The specific isoforms, their protein length and the UniProt accession numbers selected were as follows: α-syn isoform 1 (140 aa, P37840), TDP-43 isoform 1 (414 aa, Q13148) and tau 2N4R isoform (441 aa, P10636-8). To design library peptides, overlapping fragments were chosen in a tiling approach, generally using a length of 18 amino acids and 5 amino acid overlap between fragments. In some cases, highly acidic regions such as 105 – 140 in α-syn, containing many Asp and Glu residues, would have yielded peptides bearing too much negative charge; these fragments were designed with shorter overlapping fragments of length 7 – 16 amino acids. To avoid oxidation of Cys residues that can form non-natural aggregates, all Cys sites were mutated to Ala. Finally, additional basic and spacing residues were appended to the N- and C-termini of each peptide to produce peptides suitable for liquid chromatography tandem mass spectrometric (LC–MS/MS) analysis. The final substrate library contained an equimolar mixture of 77 peptide substrates with a maximum length of 24 amino acids, with the sequences provided in Table S1.

MSP-MS reactions were prepared with proteases at 1–70 nM concentration with a library concentration of 500 nM for each peptide (except CTSF at 1 µM) and incubated at 37 °C. Buffers were sodium acetate buffer (50 mM) containing 5 mM DTT and 1 mM EDTA for pH 4.5 and pH 5.5 or sodium citrate (50 mM) containing 5 mM DTT and 1 mM EDTA at pH 3.5. Reactions were monitored at two time points in an end point screening format. Each aliquot taken at a given time point was immediately desalted with C18 zip tips (Millipore-Sigma) and then freeze-dried. Samples were re-suspended in 0.1% formic acid in HPLC-grade water for LC–MS/MS analysis.

Reaction conditions for each protease followed manufacturer recommendations for pre-activation and then were assayed at the concentrations and pH buffer conditions mentioned in Table S2. For CTSA, a matched CTSL-only sample was also prepared for this reaction for use as a negative control in cleavage identification. The final CTSA reaction contained 41 nM CTSA with a background of 8.25 nM CTSL, treated with E64 (440 nM). The matched reaction contained only CTSL and E64.

### Mass spectrometry

Peptide sequencing by LC–MS/MS was performed on a QExactive Plus mass spectrometer (Thermo) equipped with a nanoACQUITY (Waters) ultraperformance LC (UPLC) system and an EASY-Spray ion source (Thermo). Reversed-phase chromatography was carried out with an EASY-Spray PepMap C18 column (Thermo, ES800; 3 μm bead size, 75 μm by 150 mm). Chromatography was performed at a 600-nl/min flow rate during sample loading for 14 min and then at a 400-nl/min flow rate for peptide separation over 90 min with a linear gradient of 2 to 35% (vol/vol) acetonitrile in 0.1% formic acid. Peptide fragmentation was performed by higher-energy collisional dissociation (HCD) on the six most intense precursor ions with a minimum of 2,000 counts, with an isolation width of 2.0 m/z and a minimum normalized collision energy of 25. Data were analyzed using Protein Prospector software, v.6.2.1 (http://prospector.ucsf.edu/prospector/mshome.htm, UCSF) using published methods [31]. The peptide cleavage data were then output as 8-mer sequences that spanned the P4 – P4’ sites for each verified cleavage site (Supplementary Data 2).

### Hierarchical clustering of protein level cleavages from MSP-MS

The percentage contribution of each enzyme to the total number of cleavages in each protein was calculated by dividing the number of cleavage sites for each enzyme with the sum of all enzyme cleavage sites. Since our substrates α-syn, TDP-43 and tau have different amino acid lengths, in order to compare the cleavage patterns, we next normalized the percentage contribution of enzyme cleavages for each substrate with the mean of total percentage contribution for each enzyme across all substrates. Using these mean-normalized values for each enzyme within each substrate, we then performed an unsupervised hierarchical clustering in Python using the clustermap function within the data visualization library called “seaborn” (https://seaborn.pydata.org/index.html).

### Correlation analysis of enzyme cleavage profiles

To compare the cleavage profile for each enzyme within each protein substrate, we computed the correlation matrix with the raw cleavage data obtained from MSP-MS and then plotted this matrix as a heatmap using the correlation function within seaborn in Python. The diagonal correlation matrix was then plotted in the form of heatmap.

### In silico analysis of MSP-MS data

The resulting peptide identifications via MSP-MS were assessed for specific cleavage in the enzyme-treated sample by subtracting the results from a no-enzyme control incubation. Both endopeptidase and exopeptidase cleavage sites were readily identified. Using these sites, we generated full sequence cleavage maps of α-syn, TDP-43 and tau. We also created diagrams of each cathepsin to identify which amino acids were favored at the P4’ through P4 positions of each enzyme’s specific cleavage motif, using the IceLogos algorithm (iomics.ugent.be/icelogoserver) [32]. We further validated our findings using the PROSPER protease prediction algorithm server (https://prosper.erc.monash.edu.au/) [28]. To determine which mutations might alter protease cleavage, we also conferred with the MEROPS database (https://www.ebi.ac.uk/merops/), assessing the amino acid frequency at the P4’-P4 positions, where all known cathepsins (cathepsins A, B, D, E, F, K, L, O, S, V, X and AEP) were previously found to cleave [27]. Using the results of our MEROPS and PROSPER searches as well as our experimental results, we identified regions of α-syn, TDP-43 and tau where pathogenic mutations overlapped with protease cleavage sites. Using this information, we designed fluorogenic peptides to test for our activity assays.

### Protease activity assays

Fluorescence-based protease activity assays were performed in triplicates in black, flat-bottom 384-well plates (Greiner #781,091, Fisher Scientific) using custom designed fluorescent α-syn, TDP-43 and tau peptide substrates (Genscript, Table S3). Assays were run at 37 °C for 10 h in 50 mM sodium citrate (pH 3.4), 50 mM sodium acetate (pH 4.5 and 5.5) or 50 mM phosphate buffer saline (pH 7.4) buffers containing 2 mM DTT and 1 mM EDTA. The concentration of cathepsins and fluorescence substrate in all the assays was 20 nM and 20 μM, respectively. The substrates were designed in the form of a fluorophore/quencher paired lysine conjugated with 7-methoxycoumarin-4-acetic acid (MCA) as a fluorophore on the N-terminus, and a lysine conjugated to dinitrophenol (DNP) as a quencher on the C-terminus. In addition, two arginine residues were added to all the substrates in order to increase the solubility of peptides in the buffers. When the substrate is intact, MCA is non-fluorescent due to the presence of the DNP quencher. However, when the substrate is cleaved by means of protease activity, MCA becomes unquenched and fluorescent. This MCA fluorescence, which serves a readout for substrate cleavage, was monitored as a function of time in a Tecan Infinite M Plex plate reader using excitation and emission wavelengths of 328 nm and 393 nm, respectively.

Raw MCA fluorescence data was normalized from 0–2000 using Microsoft Excel Version 16.49. Normalized data was then input into a kinetic model using Berkeley Madonna Version 10.2.8. to fit the enzyme kinetic data using the Rosenbrock Stiff method. Equations of the model were as follows:

$$\begin{array}{c}\mathrm{d}/\mathrm{dt }(\mathrm{C}1) = -\mathrm{a}*\mathrm{C}1*\mathrm{C}1-\mathrm{b}*\mathrm{C}1*\mathrm{C}\\ \mathrm{d}/\mathrm{dt }(\mathrm{C}) = +\mathrm{a}*\mathrm{C}1*\mathrm{C}1+\mathrm{b}*\mathrm{C}1*\mathrm{C}\\ \mathrm{d}/\mathrm{dt }(\mathrm{S}) = -\mathrm{k}*\mathrm{C}*\mathrm{S}\\ \mathrm{ d}/\mathrm{dt }(\mathrm{P}) =\mathrm{ k}*\mathrm{C}*\mathrm{S}\end{array}$$

C1 represented the proform of the cathepsin, C represented the mature cathepsin, S represented the full custom designed fluorescent α-syn, TDP-4, and tau peptide substrates, and P represented the cleaved substrate. Initial concentrations of the proform cathepsin and the substrate were 2 nM and 2000 nM, respectively to represent experimental concentrations. Parameters a & b represented cathepsin maturation. Parameter a represented the slow auto-activation mechanism by the proenzyme, while parameter b represented the faster rate of activation that occurs by the mature cathepsin. Initial estimates for parameter a were 0.05 and 0.15, and 0.5 and estimate for parameter b was 1.5. Parameter k represents substrate cleavage. Initial estimates for parameter k were 5.0e-4 and 0.0015. All parameters were constrained to be positive values. Normalized MCA fluorescence data was fit to variable P.

### In vitro lysosome degradation experiments

Commercially available, full-length, recombinant WT α-syn (ab51189), A53T α-syn (ab256149), WT 2N4R tau (ab199583) and N279K 2N4R tau (ab269006) were purchased from Abcam. Full-length WT TDP-43 and Q331K TDP-43 were synthesized, purified, and obtained from GenScript. Each of these proteins were incubated with 50 μg of human liver lysosomes (Xenotech #H0610.L) in a 50 mM sodium acetate (pH 4.5) buffer buffer containing 2 mM DTT and 1 mM EDTA. Each reaction was allowed to run for 30 min with samples taken at 0-, 10-, 20-, and 30-min time points. Results were then analyzed via Western blot.

### Cell-based protein half-life experiments

SH-SY5Y human neuroblastoma cells were obtained from ATCC (CRL-2266) and maintained 1:1 EMEM/F12 media (ATCC #30–2003/Thermo #11,765,062) supplemented with 10% FBS and 1% Penicillin–Streptomycin (Thermo Fisher #15,140,122). Stable lines of all 17 aforementioned constructs were generated by lentiviral infection followed by both blasticidin (5 µg/mL) and puromycin (1 mg/mL) selection. Cells were seeded at 30,000 cells per cm^2 and allowed to grow until ~ 70% confluent. To terminally differentiate into neuron-like cells, SH-SY5Y cells were then treated with retinoic acid as previously published [33]. Once differentiated, SH-SY5Y cells were treated with 1 mg/mL of doxycycline to induce expression of FLAG-tagged α-syn, TDP-43 or tau for 24 h (day -2). The following day (day -1), the media was refreshed without doxycycline. On day 0, for proteasomal inhibition studies, differentiated SH-SY5Y cells had their media refreshed and supplemented with 100 nm MG132 (EMD Millipore #474,791). Minimal cell death was observed after 5 days of this treatment (day 0—day 5). Cells were harvested by first aspirating off the media and then rinsing with PBS to remove any exogenous protein and/or cellular debris. Once rinsed, cells were detached and collected using a cell scraper. In this manner, cells were harvested at 0, 24, 48, 72, 96, and 120 h timepoints (i.e., days 0, 1, 2, 3, 4 and 5). Cells lysates were then spun down by centrifugation. Cell pellets were then again rinsed with PBS. Cells were lysed in RIPA buffer in the presence of protease inhibitors: cOmplete Protease Inhibitor Cocktail (Sigma #4,693,159,001), Pepstatin A (Sigma #PS-318), and PhosSTOP (Sigma # 4,906,837,001). Protein lysates were then quantified by means of a BCA Assay (ThermoFisher #23,227).

All iPSC lines (A53T/A53T α-syn, Q331K/Q331K TDP-43, N279K/N279K tau, and WT/WT TDP-43 [KOLF2.1] control) were obtained from the NIH’s iPSC Neurodegenerative Disease Initiative (iNDI) in association with the Jackson Laboratory [34]. IPSCs were maintained in Matrigel (Corning, #354,277)–coated plates using STEMFLEX media (ThermoFisher, #05,850). For passaging, iPSCs were detached using ReLeSR (STEMCELL Technologies, #05,872) and seeded using STEMFLEX media supplemented with 10 μM Rock inhibitor (Y-27632, STEMCELL Technologies, #72,304). Media was replaced every other day. iPSCs were differentiated into cortical neurons by infection with lentivirus particles containing doxycycline-inducible neurogenin-2 (Ngn2) and puromycin resistance as previously described [35, 36]. After infection, cells were cultured for at least 14 days to form mature iNeurons. For proteasomal inhibition studies, differentiated iNeurons had their media refreshed and supplemented with 100 nm MG132 (EMD Millipore #474,791). Cells were grown for up to 5 days under these conditions and then analyzed. Similar to the SHSY5Y experiments, minimal cell death was observed after 5 days of this treatment (day 0—day 5). With chronic MG132 treatment (i.e., beyond day 5), iNeurons did demonstrate some reduced cell viability. Cells lysates were then spun down by centrifugation. Cell pellets were then again rinsed with PBS to remove any remaining cellular debris. Cells were lysed in RIPA buffer in the presence of protease inhibitors: cOmplete Protease Inhibitor Cocktail (Sigma #4,693,159,001), Pepstatin A (Sigma #PS-318), and PhosSTOP (Sigma # 4,906,837,001). Protein lysates were then quantified by means of a BCA Assay (ThermoFisher #23,227).

### Lysosome isolations

To confirm trafficking of WT and mutant α-syn, TDP-43 and tau into the lysosomes of our SH-SY5Y cells, we performed a series of lysosome isolation experiments. SH-SY5Y cells were differentiated and treated with MG132 as previously described [37, 38]. Cells were harvested on day 14 post-differentiation and the lysosomes of these cells isolated using a gradient-ultracentrifuge based technique (Lysosome Enrichment Kit for Tissues and Cultured Cells from Thermofisher, # 89,839). Successful isolation and enrichment of lysosomes were confirmed by Western Blot using a Lamp1 antibody (Cell Signaling #9091).

### Western blotting

Western blotting was performed as previously described^34^. Briefly, 40 µg of protein were separated on 4–12% SDS-PAGE (Thermo) and then transferred onto nitrocellulose membranes (Bio-Rad). Membranes were blocked at room temperature with Odyssey Blocking Buffer (LI-COR 927–40,100) and incubated at 4 °C overnight with primary antibodies and 1 h at room temperature with appropriate fluorescent secondary antibodies (1:5000) (LI-COR). Immunoreactive bands were visualized using a LI-COR Odyssey CLx image scanner and quantified using ImageJ software.

### Statistical analysis

Details of the statistical test used for each experiment is in figure legends along with n and *p* value. All data is represented as mean ± SD. Statistical analysis was performed using GraphPad Prism 9 (GraphPad Software, La Jolla, California USA). Western blot quantifications for Figs. 6, S5, and S6 were analyzed using 2-way, repeated measures ANOVA analyses. Pearson’s correlational coefficient testing was used for the pairwise correlational analyses for Fig. 5c-e. A *p*-value < 0.05 was considered significant.

## Results

### Lysosomal proteases digest recombinant human α-syn, TDP-43 and tau in a selective fashion

**Fig. 1:**
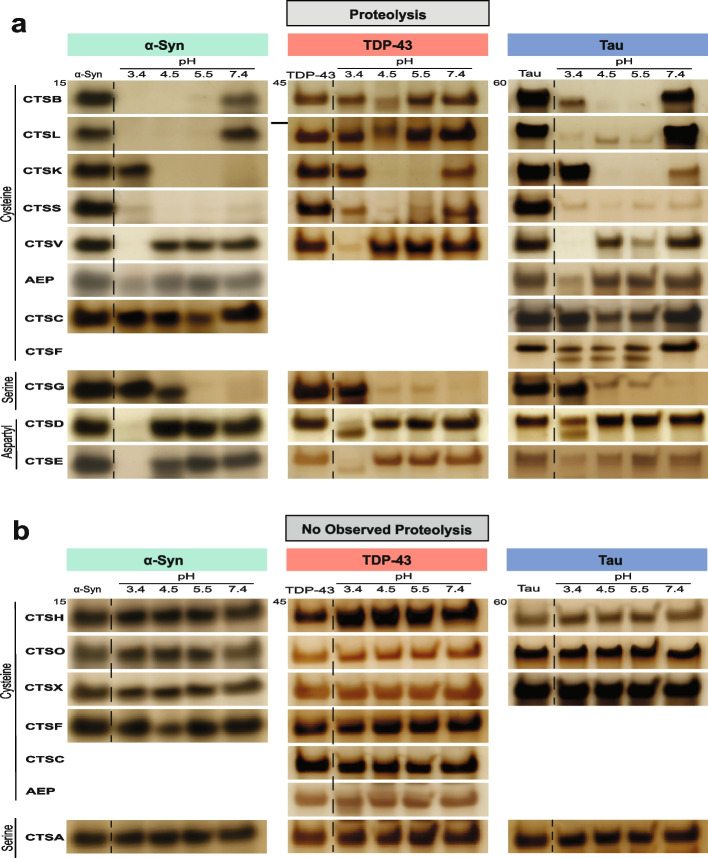
Lysosomal proteases digest α-syn, TDP-43 and tau in a selective fashion. Recombinant, human, full-length α-syn, TDP-43 or 2N4R tau (1 µg) were incubated with 1 µM lysosomal protease for 1 h at the indicated pH. Reactions were subjected to SDS-PAGE and gels visualized by silver stain. Gels are representative of *n* = 3 independent replicates. Protein and protease combinations are organized according to those in which the protease can cleave (**a**) or cannot cleave (**b**) the protein. Full-length gels are shown in Supplementary Data 1 and control reactions for those proteases that cannot cleave any of the substrates are shown in Figure S1

Although prior studies have demonstrated that α-syn, TDP-43 and tau can be shuttled to the lysosome for degradation [24-26], the specific proteases that carry out their degradation once inside the lysosomal compartment have not been fully characterized. Thus, we set out to broadly identify which lysosomal proteases are able to cleave α-syn, TDP-43 and tau via in vitro cleavage assays. Different cathepsins have distinct pH optima. Thus, to better capture the full range at which proteases can and cannot cleave these proteins we performed individual reactions of full-length, soluble α-syn, TDP-43 or tau with each protease across a broad range of pH settings (i.e., pH 3.4, 4.5, 5.5, and 7.4) (Fig. 1, Supplementary Data 1).

We first assayed the largest class of lysosomal proteases, the cysteine protease family [39]. Under our experimental conditions, cathepsins B (CTSB), L (CTSL), K (CTSK), S (CTSS), and V (CTSV) were capable of cleaving all three full-length proteins at or near their optimal pH within the span of 1 h (Fig. 1a, Supplementary Data 1). Cathepsin C (CTSC) and asparagine endopeptidase (AEP) appeared to cleave α-syn and tau moderately but not TDP-43. Interestingly, cathepsin F (CTSF) could only cleave tau in this assay (Fig. 1a, b). Most cysteine proteases cleaved α-syn, TDP-43 and tau within their known pH optima (typically between pH 4.5 to 6.5). However, CTSV, an enzyme previously observed to cleave at lower pHs than other cysteine proteases [40], appeared most active against these full-length protein substrates at pH 3.4. Intriguingly, these results suggest that CTSV in vivo may only be active against these substrates in the context of mature, highly acidified lysosomes.

Next, we tested the serine proteases, cathepsin A (CTSA) and cathepsin G (CTSG). While CTSG was able to digest all three substrates at more neutral pHs, CTSA did not demonstrate any clear protease activity against any of our substrates in this assay (Fig. 1a, b, Supplementary Data 1). Lastly, we tested the lysosomal aspartyl proteases, cathepsin D (CTSD) and cathepsin E (CTSE). CTSD has been implicated in multiple neurodegenerative diseases, including a fatal congenital form of neuronal ceroid lipofuscinosis caused by *CTSD* mutations [41]. Consistent with their known pH optima, both CTSD and CTSE digested α-syn, TDP-43 and tau only at pH 3.4, the most acidic pH tested (Fig. 1a).

Although most of the enzymes tested are endopeptidases (i.e., enzymes that can cleave peptide bonds within the middle portions of a protein substrate), cathepsins A, C, H, and X are primarily exopeptidases, hydrolyzing mainly N- or C-terminal peptide bonds and exhibiting limited endopeptidase activity. Within the context of our assay, we noted that cathepsins A, H, and X (exopeptidases) as well as the endopeptidase cathepsin O (CTSO) did not demonstrate any detectable activity against α-syn, TDP-43 or tau (Fig. 1b). To confirm the proteolytic activity of these enzymes, however, we performed fluorescence-based cleavage assays with fluorogenically-tagged casein, confirming that all enzymes are active and can cleave this universal substrate (Fig. S1).

### Multiplexed substrate profiling by mass spectrometry (MSP-MS) reveals comprehensive lysosomal protease cleavage maps of α-syn, TDP-43 and tau

**Fig. 2:**
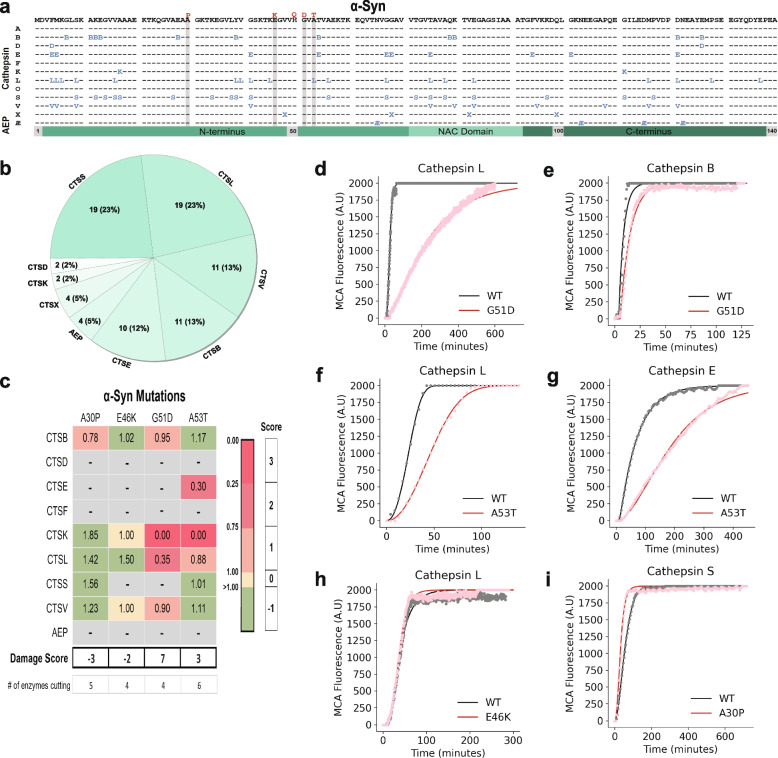
α-Syn proteolysis map and in vitro fluorescence protease assays. **a** A peptide library tiling across α-syn was incubated with individual lysosomal proteases (left). At various times and pHs, the reaction was subjected to MSP-MS to detect proteolytic cleavage sites in α-syn. The amino acid sequence of α-syn is in black letters at the top. Cleavage sites are indicated with the enzyme letter (e.g., B for CTSB) positioned at the P1 position (*e.g.* the B for CTSB is at amino acid position 7, so the cleavage occurs between positions 7 and 8). A total of 82 cleavages were found. Autosomal-dominant coding mutations associated with Parkinson’s Disease are noted in red above the α-syn sequence. Grey bars highlight amino acid mutations tested in in vitro fluorescent protease assays. **b** Pie chart demonstrating the number of cleavage sites within the α-syn sequence for each protease with the percentage of contributed cleavage sites in parentheses. **c** Table of maximal velocity (Vmax) ratios (mutant/WT), comparing protease cleavage of WT versus mutant α-syn peptides. A grey box denotes a mutation which was predicted to be "non-damaging." Mutations decreasing the Vmax of protease cleavage by 0–25% (1 point), 25–75% (2 points), and > 75% (3 points) are highlighted in light pink, dark pink, and dark red, respectively. Mutations augmenting the cleavage rate (-1 point) are highlighted in light green. Mutations with similar rate of cleavage compared to WT (0 points) are highlighted in yellow. Grey boxes denote no observed cleavage for either the WT or mutant peptide. Points were summed to derive a total “Damage Score”. **d-i** Representative curves of fluorescence generated from α-syn peptide cleavage, comparing WT and mutant peptides as labeled (*n* = 3 for all protease-substrate pairs). NAC, non-amyloid component domain; Æ, asparagine endopeptidase (AEP)

**Fig. 3:**
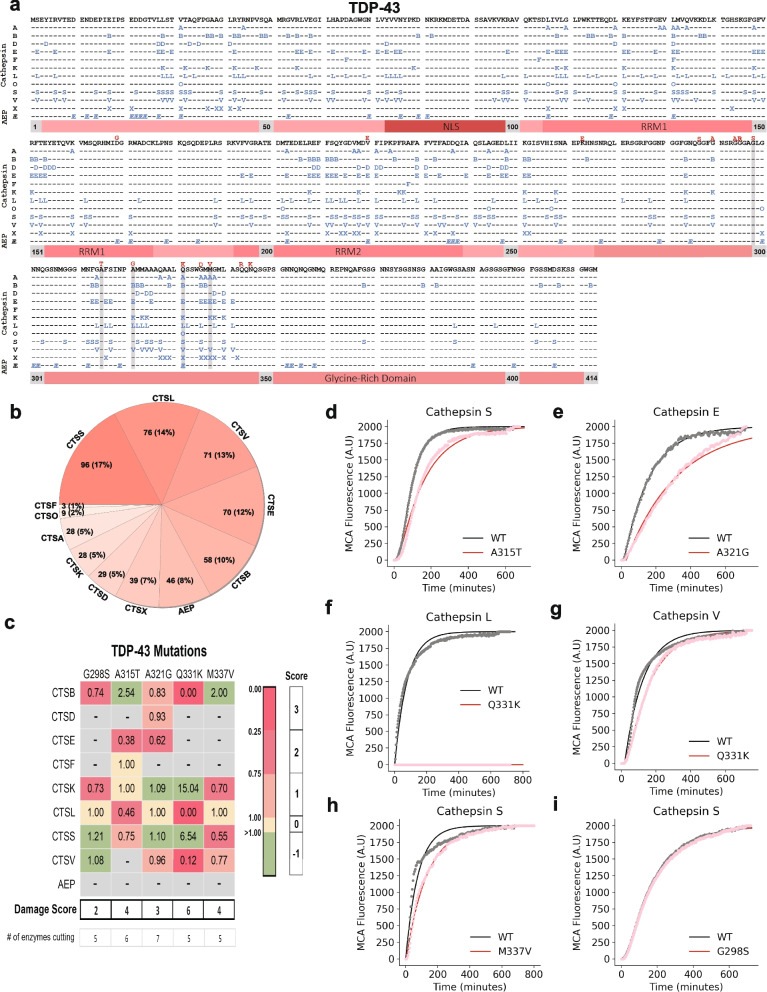
TDP-43 proteolysis map and in vitro fluorescence protease assays. **a** A peptide library tiling across TDP-43 was incubated with individual lysosomal proteases (left). At various times and pHs, the reaction was subjected to MSP-MS to detect proteolytic cleavage sites in TDP-43. The amino acid sequence of TDP-43 is in black letters at the top. Cleavage sites are indicated with the enzyme letter (e.g., B for CTSB) positioned at the P1 position (*e.g.* the B for CTSB is at amino acid position 17, so the cleavage occurs between positions 17 and 18). A total of 553 cleavages were found. Autosomal-dominant coding mutations associated with amyotrophic lateral sclerosis are noted in red above the TDP-43 sequence. Grey bars highlight amino acid mutations tested in in vitro fluorescent protease assays. **b** Pie chart demonstrating the number of cleavages sites within the TDP-43 sequence for each protease with the percentage of contributed cleavage sites in parentheses. **c** Table of maximal velocity (Vmax) ratios (mutant/WT), comparing protease cleavage of WT versus mutant TDP-43 peptides. A grey box denotes a mutation which was predicted to be "non-damaging." Mutations decreasing the Vmax of protease cleavage by 0–25% (1 point), 25–75% (2 points), and > 75% (3 points) are highlighted in light pink, dark pink, and dark red, respectively. Mutations augmenting the cleavage rate (-1 point) are highlighted in light green. Mutations with similar rate of cleavage compared to WT (0 points) are highlighted in yellow. Grey boxes denote no observed cleavage for either the WT or mutant peptide. Points were summed to derive a total “damage score”. **d-i** Representative curves of fluorescence generated from TDP-43 peptide cleavage, comparing WT and mutant peptides as labeled (*n* = 3 for all protease-substrate pairs). NLS, nuclear localization sequence; RRM1 and RRM2, RNA recognition motifs 1 and 2; Æ, asparagine endopeptidase (AEP)

**Fig. 4:**
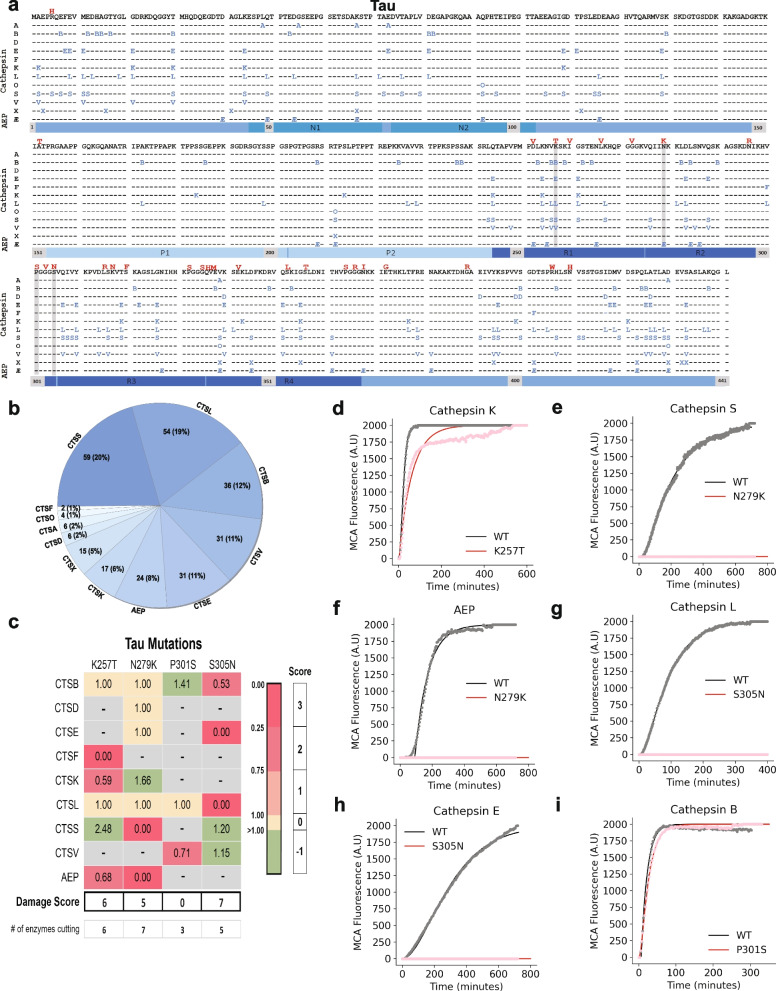
Tau proteolysis map and in vitro fluorescence protease assays*. ***a** A peptide library tiling across tau was incubated with individual lysosomal proteases (left). At various times and pHs, the reaction was subjected to MSP-MS to detect proteolytic cleavage sites in tau. The amino acid sequence of tau is in black letters at the top. Cleavage sites are indicated with the enzyme letter (e.g., B for CTSB) positioned at the P1 position (*e.g.* the B for CTSB is at amino acid position 7, so the cleavage occurs between positions 7 and 8). A total of 285 cleavages were found. Autosomal-dominant coding mutations associated with frontotemporal dementia are noted in red above the tau sequence. Grey bars highlight amino acid mutations tested in in vitro fluorescent protease assays. **b** Pie chart demonstrating the number of cleavages sites within the tau sequence for each protease with the percentage of contributed cleavage sites in parentheses. **c** Table of maximal velocity (Vmax) ratios (mutant/WT), comparing protease cleavage of WT versus mutant tau peptides. A grey box denotes a mutation which was predicted to be "non-damaging." Mutations disrupting protease cleave by 0–25% (1 point), 25–75% (2 points), and > 75% (3 points) are highlighted in light pink, dark pink, and dark red, respectively. Mutations augmenting the rate cleavage (-1 point) are highlighted in light green. Mutations with similar rate of cleavage compared to WT (0 points) are highlighted in yellow. Grey boxes denote no observed cleavage for either the WT or mutant peptide. Points were summed to derive a total “damage score”. **d-i** Representative curves of fluorescence generated from tau peptide cleavage, comparing WT and mutant peptides as labeled (*n* = 3 for all protease-substrate pairs). N1 and N2, N-terminal repeats; P1 and P2, proline-rich regions, R1-4, microtubule binding repeats 1–4; Æ, asparagine endopeptidase (AEP)

To map the specific locations at which lysosomal proteases cleave α-syn, TDP-43 and tau, we took advantage of MSP-MS, a rapid, quantitative, reproducible and unbiased method of direct substrate cleavage site identification [31]. To aid in the identification of all possible cleavage sites, we designed a custom library of seven to eighteen-residue, overlapping peptides covering the entire sequences of α-syn, TDP-43 and tau (Table S1). In contrast to previous studies which analyzed protease activity on either full-length protein or protein fibrils [42–45], we specifically chose linear peptides to avoid the possibility of steric hindrance from protein secondary structure and to better model the denatured and ultimately partially cleaved state that most soluble proteins attain within the complex, acidic, protease-filled environment of lysosomes. Moreover, each peptide was designed to generate unique fragments for tandem mass spectrometry (MS/MS) detection. The library was then incubated with individual proteases at one or more pH setpoints within their optimal range and cleavage fragments assessed at two time points (Table S2). Cathepsins G, C and H were excluded due to reportedly low neuronal expression and because the latter two require a pre-activation step that is technically incompatible with MSP-MS [31, 46]. In total, the peptide library was tested against twelve recombinant proteases which generated 920 total cleavages across the three proteins. The results of this extensive mapping campaign are shown in Figs. 2a, 3a and 4a as well as in Supplementary Data 2.

### α-Syn

At 140 amino acids, α-syn is the smallest of the three proteins assessed. In total, there were 82 cleavages in α-syn by 9 of the 12 proteases tested (Fig. 2a). In agreement with the recombinant, full-length α-syn in vitro protease assay results (Fig. 1b), cathepsins A, F and O did not cleave any of the peptides within the library. Remarkably, CTSX, a protease that could not cleave full-length α-syn, was able to digest several α-syn peptides. This highlights the advantages of utilizing short peptides in this assay, since under physiological conditions secondary structure will eventually be resolved as individual lysosomal cathepsins will ultimately see peptides cleaved by other proteases. Protease cleavage sites were evenly distributed throughout the α-syn sequence, including within the non-amyloid component (NAC) domain which forms the core of α-syn fibrils [2]. Of the proteases tested, CTSL and CTSS displayed the most cleavage sites in the α-syn peptides with CTSB and CTSV following (Fig. 2b). Of the proteases examined, CTSD has previously been shown to cleave soluble α-syn [42] while cathepsins B, K, and L are capable of cleaving α-syn fibrils [43–45]. Our results confirm CTSD as a soluble α-syn protease and substantially supplement the known lysosomal proteases that can process α-syn to include five additional: AEP as well as cathepsins E, S, V, and X.

### TDP-43

TDP-43 contains 414 amino acids. Each of the 12 proteases tested was able to cleave TDP-43 peptides in multiple locations, resulting in 553 total cleavages (Fig. 3a). Cathepsins S, L, V and E had the greatest number of proteolytic sites (Fig. 3b). Full-length, recombinant TDP-43 in solution likely retains some secondary structure [46]. As such, it was notable that cathepsins A, F, O, X and AEP, all of which did not appear to degrade full-length TDP-43 in Fig. 1, were able to cleave linear TDP-43 peptides under these MSP-MS conditions. Prior experimental data has demonstrated cleavage sites within TDP-43 for CTSL (positions 32, 341) and CTSS (position 341) [27]. Our results validated these sites and provided many more additional CTSL and CTSS cleavage sites. Notably, protease processing sites were not uniformly distributed across TDP-43. One region replete with protease sites, amino acids 306–378, forms the densely packed common core of TDP-43 fibrils [46, 47]. In contrast, other regions within TDP-43 exhibited a relative paucity of cleavage sites, including within the carboxy (C)-terminus. Regions with a relative paucity of protease processing sites, such as the C-terminal glycine rich domain (amino acids 351 to 414) did contain AEP sites. This is consistent with prior reports that AEP exhibits relatively distinctive, non-overlapping cleavage recognition sequences compared to other proteases [33, 48].

### Tau

The longest adult isoform of tau (2N4R) contains 441 amino acids. Other than previously reported cleavage by CTSD and CTSS, little is known regarding lysosomal proteases that can digest tau [49, 50]. All twelve of the proteases tested cleaved tau peptides for a total of 285 cleavages (Fig. 4a). Similar to TDP-43, cathepsins A, O and X, which did not cleave full-length recombinant tau, exhibited a few sparse cleavages of tau peptides. CTSS and CTSL displayed the highest numbers of tau cleavage sites with cathepsins B, V, E and AEP following (Fig. 4b). Also like TDP-43, protease cleavage sites within tau clustered together. Portions of tau, encompassing the proline rich P1 and P2 domains, contained relative few proteolytic sites, with residues 131 to 170 demonstrating no cleavage sites in this assay. The region from residues 306 to 355 in tau makes up the structural core of paired helical and straight filaments found in AD [4]. Again in alignment with TDP-43, this region was relatively rich in cathepsin cleavage sites.

### Proteases exhibit distinctive abilities to process α-syn, TDP-43 and tau

**Fig. 5:**
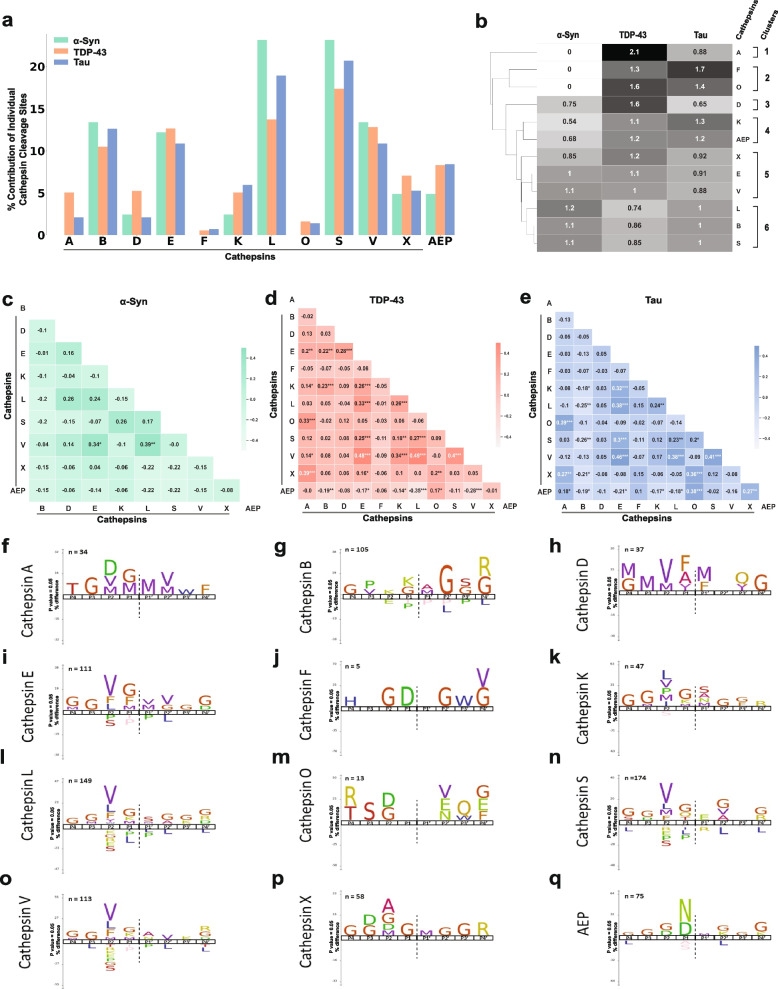
Lysosomal proteases exhibit distinctive abilities to process α-syn, TDP-43 and tau. **a** Comparison of the number of cleavage sites within α-syn, TDP-43 and tau for each lysosomal protease relative to total number of cleavage sites. **b** Hierarchical clustering analysis demonstrating the relative affinity of proteases for α-syn, TDP-43 or tau. Clusters identified are designated to the right. **c-e** Pairwise correlation analyses with significance values (*p*-values) of unique versus redundant activity between lysosomal proteases for α-syn (C), TDP-43 (D) and tau (E). A positive score suggests more correlation and a negative score lower correlation between protease cleavage sites. **f-q** The iceLogo output for each of the serine (F), aspartyl (G-H) and cysteine (I-Q) proteases demonstrating the frequency of particular amino acids at the P4 – P4’ positions of each protease recognition motif within α-syn, TDP-43 and tau. Amino acids that were more frequently seen are above the horizontal axis and those that were less frequently seen are below the horizontal axis. The cleavage site is indicated with a vertical hatched line. *p < 0.05, **p < 0.01, or ***p < 0.001

Having mapped the lysosomal proteolytic cleavage sites in α-syn, TDP-43 and tau, we next wondered if any of the proteases exhibited preferences for one of these substrates over the others. This prospect was of significant interest as cathepsins exhibit age-dependent, brain-region and cell-type specific variability in expression and activity [51–53]. Thus, preferential behavior of certain lysosomal proteases towards α-syn, TDP-43 or tau could contribute to selective neuronal vulnerability observed in different pathologies (*e.g.*, PD versus ALS). Therefore, we performed comparative analysis of the proteases in regards to proteolysis of α-syn, TDP-43 and tau. To do so in proteins of different sizes, we normalized the number of cathepsin cleavages for each substrate relative to their amino acid number. Among these proteases, we found that two cysteine cathepsins, CTSL and CTSS, demonstrated the highest proportion of cleavage sites in α-syn, TDP-43 and tau (Fig. 5a). Cathepsins B, E, V and AEP also exhibited significant activity with numerous sites of cleavage identified. Comparing the aspartyl proteases, CTSE exhibited significantly more cleavage sites than CTSD. Notably, cathepsins A, F and O demonstrated relatively few sites of cleavage within tau and TDP-43 and no cleavage sites within α-syn.

Co-pathologies are often found in neurodegenerative diseases, especially with increasing age, thus similarities or differences between cathepsin substrate preferences may be important in this process [54]. To assess which proteases are more or less alike in their ability to cleave α-syn, TDP-43 and tau, we performed hierarchical clustering [55]. This clustering analysis revealed up to six distinct clusters of cathepsins based on their relative preferences towards the three substrates (Fig. 5b). The first two clusters consisted of cathepsins A, F and O, which could not cleave α-syn. Cathepsins X, E and V formed a cluster that exhibit relatively even distributions of cleavages within α-syn, TDP-43 and tau. A cluster including cathepsins L, B and S had a slight preference for α-syn cleavage while in contrast, CTSK and AEP favored TDP-43 and tau over α-syn. Lastly, cathepsins A and D rather heavily favored TDP-43, and CTSF preferred tau. This knowledge would be important if these proteins were to be targeted for enhanced degradation through cathepsin upregulation.

Because little is known about redundant versus non-redundant activity among these proteases, we also searched for similarities among cathepsins within the cleavage site profile of each substrate. We performed this survey since it would be highly relevant if upregulating protease activity were to become potential treatments in neurodegeneration. To this end, we performed pairwise correlational analyses. This analysis revealed that across the three substrates, the cleavage profiles of the aspartyl protease cathepsin E and the cysteine proteases cathepsins L and V were positively correlated with one another (Fig. 5c-e). Cathepsin X was also positively correlated with CTSA and CTSO for TDP-43 and tau. Somewhat surprisingly, CTSB showed either minimal or a negative correlation with most other cathepsins in α-syn and tau. This is fascinating as CTSB has previously been shown to cleave similar sequence motifs that of other cysteine proteases, such as CTSL and CTSS, when profiled with more generalized, screening peptide libraries [29]. In contrast to CTSB, however, AEP demonstrated an expectedly negative correlation pattern with the other tested cathepsins, as AEP is known to be highly specific for the endoproteolytic cleavage of asparagine while most other proteases do not favor asparagines at the P2 through P2' positions [26]. Overall, this analysis suggests that certain cathepsins could potentially work redundantly to degrade α-syn, TDP-43 or tau. In contrast, other cathepsins, such as CTSB and AEP, cleave relatively unique regions in these proteins. Thus, if CTSB or AEP activity were lost, proteolytic cleavage in α-syn, TDP-43 and tau may be affected in a manner that is both similar and not easily compensated by other proteases. Intriguingly, this finding raises the possibility that the molecular basis for disease co-pathology (e.g., findings of synucleinopathy plus tauopathy in the same brain regions) could be due to the early or preferential loss of CTSB or AEP activity.”

### A subset of neurodegenerative disease-associated mutations is predicted to disrupt lysosomal protease cleavage

While individual proteases have preferred amino acid recognition sequences, certain positions within their recognition sequence tend to exert outsized importance. Protease cleavage sequences are expressed based on the positions relative to the cleavage site, with (from left to right) P4 to P1 found on the amino (N)-terminal side of the cleavage site and P1’ to P4’ found on the C-terminal side. When expressed in this fashion, many of the cysteine proteases (e.g., cathepsins L and V) prefer hydrophobic residues at P2 [56] while AEP prefers asparagine at P1 [57]. Serine proteases rely heavily upon the P1 position for recognition [58]. Aspartyl proteases like CTSD and CTSE strongly prefer hydrophobic residues at P1 and P1’ [27].

Having generated cathepsin cleavage maps for α-syn, TDP-43 and tau, we overlaid the known disease-associated variants onto the maps (Figs. 2a, 3a and 4a). In several cases, disease-associated mutations appeared in close proximity to protease cleavage sites and were predicted to alter amino acids important for protease recognition [59]. For example, the TDP-43 A315T mutation is positioned at P1’ for a CTSE cleavage site and is predicted to make cleavage less favorable, since threonine is polar and not hydrophobic like alanine is at this position (Fig. 3a). Similarly, the N279K mutation on tau removes a critical asparagine from a P1 cleavage site position for AEP (Fig. 4a). We thus asked if neurodegenerative disease-associated mutations could directly alter the efficiency of proteolytic cleavage by lysosomal proteases.

Since it was not feasible to test every mutation against every protease, we utilized two approaches to identify a subset of mutations mostly likely to abrogate cathepsin cleavage. First, we performed in silico comparisons of WT versus mutant protein sequences in the PROSPER database (https://prosper.erc.monash.edu.au/) [28]. This analysis generated potential cleavage sites as well as a rank order set of mutations predicted to change proteolytic cleavage (Fig. S2, Table S3). Second, we used the MSP-MS data to assess whether specific amino acids are preferentially recognized by certain proteases, specifically within the sequences of α-syn, TDP-43 and tau. To do so, we input the MSP-MS cleavage sites into the iceLogo algorithm (iomics.ugent.be/icelogoserver), which uses probability theory to identify conserved patterns in proteins [32]. The iceLogo output allowed visualization of favorable and unfavorable amino acids at each of the protease recognition motif positions (P4 to P4’) (Fig. 5f-q). Results of this analysis suggested that the amino acid preferences in α-syn, TDP-43 and tau for several of the proteases, specifically cathepsins E, L, S and AEP, were similar to previously published iceLogo maps [29, 31, 60] (Fig. 5i, l, n and q). However, CTSB showed sequence preferences within α-syn, TDP-43 and tau that diverged from current notations [29] (Fig. 5g).

From the PROSPER and iceLogo analyses, we curated a set of eleven potentially “damaging” disease mutations to test (three in α-syn, four in TDP-43 and three in tau) to test for altered proteolytic cleavage activity as well as non-damaging “control” mutations with lower probability of altering protease cleavage activity (Table S3). We then generated WT and mutant peptides encompassing these regions (Table S4). Each peptide was labelled with a coumarin-based (MCA) fluorophore and a fluorescence quencher on the N- and C-termini, respectively. The peptide substrates were thus quenched at baseline and only revealed fluorescence upon cleavage by a protease. We incubated the peptides with individual proteases and monitored the fluorescence generated over time. Given the nature of the assay, we limited our testing to the primary endopeptidases, cathepsins B, D, E, F, K, L, S and V as well as AEP.

Using this activity assay, the maximal velocities (Vmax) at which each enzyme cleaved the WT and mutant peptides were calculated (Fig. S3). Using these values, the rates of WT versus mutant cleavage were compared and ratios (mutant over WT) were generated. In this type of in vitro protease approach, every cathepsin would not be expected to cleave every peptide. Indeed, we found that each WT peptide was cleaved by three to seven of the nine proteases tested (Figs. 2c, 3c, 4c and Fig. S3). Mutant peptides were oftentimes cleaved similarly to wild-type peptides. However, in other cases they were cleaved more slowly or not at all. Rarely, mutant peptides were also cleaved more quickly than wild type. Examples for α-syn, TDP-43 and tau are described below and shown in Figs. 2c, 3c and 4c.

To better quantify the summed impact of these changes over many different proteases, we assigned “damage points” for each instance in which a mutation decreased a protease’s Vmax for peptide cleavage by 0–25% (1 point), 25–75% (2 points) or greater than 75% (3 points). In contrast, mutations that increased the rate of cleavage of mutant peptides were given a score of -1 point. By summing the point values, we were able to calculate a “Damage Score” for each mutation (Figs. 2c, 3c and 4c). Fascinatingly, each of the mutations predicted to be disruptive demonstrated positive scores of 3 to 6 while the control mutations (those disease mutations not predicted to be damaging to proteolytic cleavage) exhibited neutral or negative scores.

For α-syn, in silico predictions suggested that G51D and A53T would be damaging to proteolytic cleavage while A30P and E46K would not be. When α-syn G51D was tested via this in vitro activity assay, the cysteine proteases CTSB, CTSL, CTSK and CTSV cleaved the mutant α-syn G51D peptide less efficiently than the WT peptide for a total Damage Score of + 7 (Fig. 2c-e). The A53T mutation also diminished lysosomal protease cleavage by CTSK, CTSL and CTSE, resulting in a Damage Score of + 3 (Fig. 2c, f and g). The α-syn A30P and E46K mutations, which in silico analysis suggested would not be damaging, were also tested in vitro. Every protease capable of cleaving the wild-type peptides were also able to cleave the mutant peptides at similar or faster rates, with the single exception of CTSB cleavage of the A30P peptide (Fig. 2c, h and i). Overall, this analysis revealed that the A30P and E46K α-syn mutations had negative Damage Scores (of -3 and -2, respectively).

Disease-associated mutations in TDP-43 also affected proteolytic cleavage. TDP-43 A315T is in proximity to many protease cleavage sites (i.e., cathepsins A, B, L, S and V). When tested within our in vitro protease assays, this mutation decreased CTSE, CTSL and CTSS cleavage of the mutant peptide (Fig. 3c, d). Notably, A315T disrupts the important P1’ position for CTSE; and likely as a consequence, CTSE cleavage of the mutant A315T peptide was substantially diminished (Fig. 3c). TDP-43 A321G also decreased CTSE’s Vmax while more modestly diminishing CTSB, CTSD and CTSV activity (Fig. 3c and e). The Damage Score for A315T was + 3. The TDP-43 Q331K mutation is proximal to many cleavage sites and, when tested, completely abrogated CTSL and CTSV cleavage compared to WT (Fig. 3f and g). Similarly, the Q331K mutation also decreased CTSV cleavage significantly. Thus, Q331K had the highest Damage Score (of + 6) in TDP-43. The TDP-43 M337V mutation also has the potential to impact many proteases and indeed diminished the activity of CTSK, CTSS and CTSV (Fig. 3c and h). In contrast, the TDP-43 G298S mutation occurs within an area of TDP-43 relatively devoid of observed protease cleavage sites. Interestingly, this mutation exerted generally balanced effects on protease cleavage, as CTSB and CTSK had decreased cleavage while CTSS and CTSV had an increased rate of cleavage (Fig. 3c and i). It also had the lowest Damage Score (of + 2), consistent with in silico predictions.

Finally, tau mutations that were predicted to interfere with protease cleavage also dramatically impaired protease activity against mutant but not control peptides. CTSF, CTSK and AEP exhibited dramatically slower cleavage of the K257T mutant peptide (Fig. 4c and e). The tau N279K mutation, by removing an asparagine at an AEP P1 site, fully extinguished cleavage by AEP (Fig. 4f) as well as CTSS (Fig. 4e). CTSK cleavage, by contrast, was increased (Fig. 4c). Interestingly, the S305N mutation dramatically impacted CTSE (Fig. 4g), CTSL (Fig. 4h) and CTSB (Fig. 4c) activity. Our control “non-damaging” mutation, P301S, for which MSP-MS found limited local protease cleavage sites, demonstrated faster cleavage by CTSB that was balanced by slower cleavage of CTSV compared to WT. This is of interest as P301S is a mutation well-known to rapidly promote tau aggregate formation [61]. Thus, the minimal effect exerted by the P301S mutation on lysosomal proteolysis, as well as its low Damage Score (of + 2), suggests that P301S pathogenicity is not via altering lysosomal proteolysis.

### Disease mutations alter α-syn, TDP-43 and tau protein half-life in neuronal lysosomes

**Fig. 6:**
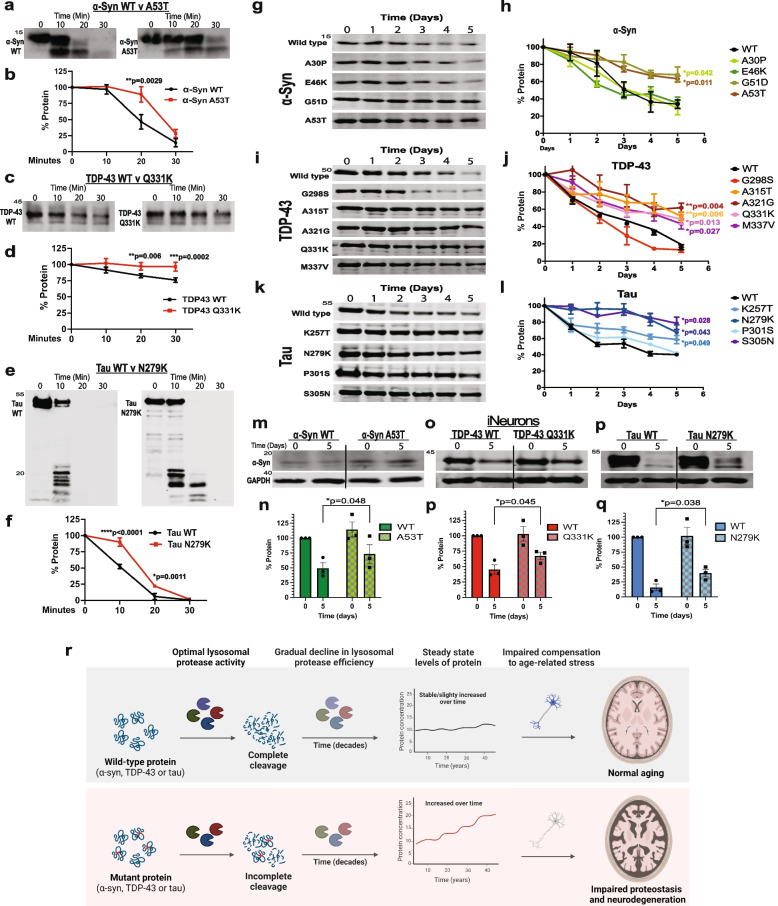
Pathogenic mutations prolong α-syn, TDP-43 and tau half-life. **a-f** Recombinant, full-length WT or A53T α-syn (**a**, **b**), WT or Q331K TDP-43 (c, d), and WT or N279K tau (**e**, **f**) was incubated with 50 μg of human lysosome extract for 30 min. The reaction was subjected to Western blot and results quantified. **g-l**, Differentiated SH-SY5Y cells expressing inducible FLAG-tagged WT or mutant α-syn (**g**, **h**), TDP-43 (**i**, **j**) or tau (**k**, **l**) were treated with doxycycline for 24 h to induce protein expression. Doxycycline was removed (t = 0 days), MG132 (100 nm) was added to inhibit proteasomal degradation and lysates were collected at each time point as indicated. Samples then underwent SDS-PAGE and anti-FLAG Western blotting (*n* = 3 for all cell-lines tested). Samples were normalized for quantification using GAPDH as a loading control. Representative images showing the gradual clearance of of WT or mutant protein over time are shown in **g**, **I** and **k** with quantification of three independent replicates shown in (**h**, **j**) and (**i**). **m-q** Control or mutant iNeurons were generated as indicated, and then exposed to MG132 treatment (100 nm) for 5 days. Lysates were collected at day 0 and day 5 and underwent SDS-PAGE and anti- α-syn, anti-TDP-43, or anti-tau Western blotting (*n* = 3 for all cell-lines tested). Samples were normalized for quantification using GAPDH as a loading control. Representative images demonstrating WT or mutant protein over time are shown in k with quantification of three independent replicates shown in (**l**). **r** Proposed model for how mutations in α-syn, TDP-43 and tau can gradually increase steady state levels of protein over decades to predispose to impaired proteostasis, protein aggregation and neurodegeneration. *p < 0.05, **p < 0.01, or ***p < 0.001

Because the initial in vitro activity assays only tested proteases individually, we next wanted to assess the impact of these α-syn, TDP-43 and tau mutants in presence of a more physiologic, complex, lysosomal protease mixture. To this end, we compared the degradation of full-length, recombinant WT versus mutant versions of α-syn, TDP-43 or tau when exposed to commercially available, isolated human lysosomes. We incubated each reaction over 30 min with samples taken at 0-, 10-, 20-, and 30-min time points. Intriguingly, the α-syn A53T, TDP-43 Q331K and tau N279K mutant proteins all exhibited significant resistance to lysosomal degradation under these conditions, compared to their WT counterparts (Fig. 6a-f). These results demonstrate that even when full-length mutant proteins are exposed to a full complement of lysosomal proteases, the mutations still significantly delayed proteolytic cleavage.

We next moved to neuronal cell models to validate which, if any, mutations impacted lysosomal proteolysis of α-syn, TDP-43 and tau in a more physiological setting. First, we used SH-SY5Y cells, a neuroblastoma cell line that can be terminally differentiated into a neuron-like state with neurite growth and expression of neuronal markers [62, 63]. We generated stable cell lines in which FLAG-tagged, full-length WT or mutant α-syn, TDP-43 or tau could be inducibly expressed. We chose an inducible system to avoid, as much as possible, the negative effects of chronic protein overexpression on autophagy, as well as to mitigate the impact of these known disease mutations on cellular health [63].

Following neuronal differentiation, we treated SH-SY5Y cells with doxycycline to induce α-syn, TDP-43 or tau expression for one day, then removed doxycycline and measured the rate of protein clearance. We observed a relatively physiological expression profiles and observed minimal leakiness of these constructs (Fig. S4). To isolate lysosomal activity, we inhibited the proteasome with 100 nm MG132 and confirmed the compensatory increase in autophagic flux that has been previously seen with this inhibitor [37] (Fig. S5). We chose 100 nm of MG132 because this dose was sufficient to inhibit the proteasome while also being relatively non-toxic and below the threshold to sufficiently impair the activity of calpains and other cytosolic proteases. We then compared the clearance of WT α-syn, TDP-43 and tau to their mutant counterparts (Fig. 6g-l, Fig. S6). For α-syn, the high Damage Score G51D and A53T mutations significantly increased protein half-life while the low Damage Score A30P and E46K mutations were similar to WT (Fig. 6g, h). Similarly, TDP-43 A315T, A321G, Q331K and M337V mutations as well as tau K257T, N279K and S305N mutations all had high Damage Scores and significantly prolonged the half-life of their substrates (Fig. 6i-l). This is in contrast to TDP-43 G298S and tau P301S mutations, which had low Damage Scores and did not extend protein half-life compared to WT. Overall, we noted consistent alignment of the Damage Scores calculated from the in vitro fluorogenic protease assays and the results from these cell-based protein half-life studies.

To discriminate the effects of protease resistance from the possibility of lysosomal trafficking disruptions by these mutations, we next performed a set of lysosome isolation experiments. In doing so, we confirmed that each of these proteins were being trafficked into lysosomes competent for chaperone-mediated autophagy at similar rates and observed similar trends in the protein levels of the lysosomal fractions as that observed in the full lysates (Fig. S7).

Finally, to mitigate the potentially confounding effects of plasmid-derived, ectopic protein expression, we performed experiments in iPSC-derived cortical neurons (iNeurons). Through the NIH’s iPSC Neurodegenerative Disease Initiative (iNDI), we obtained 3 CRISPR-edited, homozygous mutant lines, α-syn A53T, TDP-43 Q331K, and tau N279K, as well as their parent isogenic WT iPSC line [34]. These all represented mutations with high Damage Scores in the in vitro protease assay and that extended half-life in the SH-SY5Y cells. Using the protocol from Zhang et al. [35], we generated iPSC-derived cortical neurons (i.e., iNeurons) from each of these lines. The iNeurons were differentiated for 14 days and then treated with MG132 to isolate lysosomal proteolysis. At day 0 (14 days after differentiation but before MG132 treatment), steady state intracellular levels of the mutant α-syn, TDP-43 or tau in these iNeurons were similar or trended towards being slightly increased compared to WT (Fig. 6m-q, day 0). Only protein from cell lysates was measured therefore we cannot rule out that additional protein was secreted. After 5 days of MG132 treatment, autophagy induction resulted in a reduction of both WT and mutant α-syn, TDP-43 or tau levels. However, mutant α-syn, TDP-43 and tau levels persisted compared to WT in these iNeurons (Fig. 6m-q, Fig. S6), suggesting that lysosomal proteolysis of the mutant proteins were disrupted.

## Discussion

As α-syn, TDP-43 and tau are all intrinsically disordered proteins that are found in disease inclusions and whose mutations are causally linked to neurodegeneration [64], understanding the full metabolism of α-syn, TDP-43 and tau, including the specifics sites of proteolytic cleavage and the cathepsins responsible, holds particular relevance in neurodegenerative disease. To do this, we first catalogued the individual roles of each of the lysosomal proteases cleaving at specific amino acid positions within α-syn, TDP-43 and tau at various times and pH values, thereby generating a resource for future studies of lysosomal protease biology. We then demonstrated that certain “damaging” disease mutations dramatically altered proteolytic efficiency in in vitro assays and extended protein half-life in neuronal models.

Although we did observe a significant degree of redundancy in the activity of the lysosomal proteases on the degradation of α-syn, TDP-43 and tau, we were surprised to find that 1) certain proteases, such as CTSB and AEP, had relatively specific non-redundant activity and 2) certain mutations disrupted multiple proteases and likely undercut a significant degree of the redundancy present among the lysosomal proteases. Our correlation analyses also raise the question of whether certain cathepsins are regulated in a coordinated fashion, such as through the TFEB-mediated CLEAR pathway or via an alternative mechanism, in response to these specific lysosomal substrates [65].

While the increased steady-state levels of mutant α-syn, TDP-43 and tau were modest in the cell-based assays, these assays spanned only a few days. In some respects, it is remarkable that point mutations could detectably alter steady state levels of α-syn, TDP-43 and tau at all. Over weeks, years and decades, the impact of half-life extending mutations in α-syn, TDP-43 or tau would likely be compounded. They could be further exaggerated by age-associated declines in lysosome function [66–68] or through similar age-related slowdowns in the ubiquitin–proteasome system [69] that were previously able to handle (or even potentially enhance) the clearance of these mutant substrates through compensatory upregulation. Thus, while the accumulation of mutant α-syn, TDP-43 or tau in this study was rather small, when properly contextualized over decades, they could significantly impact protein homeostasis and lead to neurodegeneration (see model in Fig. 6r).

This model complements and builds upon the idea that certain disease mutations can increase steady state levels of protein directly by enhancing fibril formation while others, like the ones we identify here, do so indirectly via impaired lysosomal degradation. As such, aberrant protein homeostasis and a steady state increase in these proteins may be the unifying feature of these diseases. As terminally differentiated cells that cannot dilute protein concentrations through cell division, neurons have developed many cooperative strategies to deal with aberrant protein homeostasis (*e.g.,* chaperone upregulation, proteasome and autophagy induction, lysosomal secretion, ER and mitochondrial unfolded protein responses, aggregate formation, etc.). As such, it may only be when these compensatory mechanisms are exhausted that neurodegeneration occurs.

Our results highlight the lysosomal proteases as significant contributors to familial PD, FTD, ALS and AD pathobiology that could be differentially targeted for disease-specific therapeutic purposes. While autosomal dominant mutations in α-syn, TDP-43, and tau are causal for only a minority of neurodegenerative disease cases, they nevertheless can provide key insights into the development of sporadic forms of neurodegeneration, as these diseases are also associated with α-syn, TDP-43, and/or tau aggregation but yet occur in the absence of mutations. Our findings also suggest that unique coding single nucleotide polymorphisms (SNPs) impairing the proteolytic cleavage of these proteins in the lysosome could play a role in sporadic cases as well. The practical and disease implications of these findings are multifold. First, they provide a molecular basis by which disease mutations in *SNCA, TARDBP* and *MAPT* that do not promote protein self-association can potentially extend half-life, leading to accumulation and subsequent aggregate formation. Second, the specificity of protease-substrate relationships suggests a possible role for the lysosome in selective neuronal vulnerability, as protease types and levels differ between neuronal sub-types and brain regions [51] and could thereby predispose to α-syn, TDP-43 or tau accumulation. Third, as cleavage site-rich regions of TDP-43 and tau overlapped with what have recently been demonstrated to form the core of TDP-43 fibrils and tau filaments, our findings suggest that inefficient lysosomal proteolysis may precede and contribute to inclusion formation. Finally, this approach has uncovered distinctive relationships between certain proteases and α-syn, TDP-43 or tau that could elucidate both genetic and sporadic forms of neurodegenerative disease. For example, the preference of CTSD for TDP-43 (Fig. 5b) takes on additional meaning when connected to the knowledge that CTSD activity is promoted by the FTD-associated protein progranulin [70–72] and progranulin haploinsufficiency leads to FTD with TDP-43 inclusions [73, 74]. Similarly, the proteases that cleaved TDP-43 and tau in our study were more similar than those that cleaved α-syn. This may explain why TDP-43 and tau pathology co-exist in certain cortical regions whereas α-syn pathology, at least early in disease, tends to be more regionally distinct [75]. Finally, these discoveries also give rise to a novel mechanism by which both autosomal dominant mutations and coding polymorphisms in α-syn, TDP-43 and tau could contribute to disease pathogenesis by interfering with lysosomal clearance.

Although our efforts were designed to be comprehensive, there are limitations to our study. Moreover, while the MSP-MS and in vitro fluorescent protease assays are complimentary (balancing sensitivity and specificity), on occasion we observed slight discrepancies. For example, in the in vitro protease assay CTSB cleaved the TDP-43 fluorescent peptides spanning the 294–302 region, which we did not observe in MSP-MS. This is likely because the MSP-MS results were limited to two collection time-points, which may miss both the earliest cleavages as well as very late events, although the latter may be less relevant given the multi-protease environment inside the lysosome. Additionally, cell-based experiments were limited to relatively short time intervals and involved differentiated neuronal cells which lack certain aging-hallmarks and whose age-related declines in proteasome function had to be simulated using a proteasomal inhibitor. Future work should address longer-term, in vivo contexts (possibly within organoid or animal experiments), where the association of end-stage disease hallmarks, such as protein aggregates, in response to cathepsin inhibition can be studied. The contributions of post-translational modifications, which can impact both lysosomal targeting and proteolytic processing [76], could also be assessed in the future. Lastly, developing and testing the efficacy of lysosomal targeted protease enhancers as therapies in human neurodegenerative diseases represent important future translational goals.

## Conclusion

This study comprehensively identifies the arsenal of lysosomal proteases that degrade α-syn, TDP-43 and tau, demonstrating that these proteases have highly specific cleavage patterns and optimal pH settings. This work also provides compelling experimental evidence that certain pathogenic mutations in each of these three proteins can disrupt this lysosomal protease activity, prolong protein half-life, and increase steady state levels in neuronal cell models. Fascinatingly, this novel, alternative mechanism by which disease-causing mutations in α-syn, TDP-43 and tau may lead to neurodegeneration also appears to be shared. These findings therefore enhance our understanding of α-syn, TDP-43 and tau metabolism as well as their impact on normal protein homeostasis and neurodegenerative disease pathobiology. In the future, this work could be leveraged to promote neuronal health by upregulating specific lysosomal proteases.

## Supplementary Information

**Additional file 1: Figure S1.** Proteases that do not cleave α-syn, TDP-43, or tau are active against a fluorogenic casein substrate. Fluorogenic protease activity assays using casein at pH 4.5 was performed on all enzymes that do not cleave α-syn, TDP-43, or tau. The values obtained were normalized to timepoint t = 0 and plotted relative to activity. All enzymes, including both cysteine and serine proteases, show activity against the optimized fluorogenic peptides compared to the substrate only control. **Figure S2.** PROSPER analysis predicts multiple cathepsin cleavage sites with α-syn, TDP-43, and tau. Cathepsins Gand K, the lysosomal proteases available in the PROSPER database, demonstrate many sites of cleavage throughout the α-syn, TDP-43, and tau substrates. Yellow highlighted amino acids represent the P1 site of predicted CTSK cleavage. Light blue highlighted amino acids represent the P1 of predicted CTSG cleavage. **Figure S3.** Cathepsin activity assay Vmax tables of α-syn, TDP-43, and tau wild type versus mutant substrates. a, α-Syn, b, TDP-43, and c, tau. Vmax values are listed in nM/min. An arbitrary value of 2000 value was given to protease-enzyme substrate interactions that were too fast to be modeled. **Figure S4.** Doxycycline-induced expression of mutant α-syn, TDP-43, and tau in differentiated SHSY-5Y cells. a, α-Syn; b, TDP-43; and c, tau. Western blot images demonstrating induced expression of each FLAG-tagged protein by our plasmid constructs with 24 h of doxycycline treatment results in measurable protein whose half-life can be readily quantified over days. These results also demonstrate minimal leakiness of these constructs in the absence of doxycycline. **Figure S5.** Proteasomal inhibitor, MG132, upregulates markers of autophagy. a., Western blot images demonstrating that Lamp1, cathepsin D, LCBIIb, and TFEB are all upregulated after MG132 treatment at 24, 72, and 120 h, compared to the no treatment condition. b., Quantification of the results of A. Experiments were performed in triplicate. **Figure S6.** Full Western blots of differentiated SH-SY5Y and iNeuron protein half-life experiments. a. Western blot images demonstrating induced expression of each FLAG-tagged protein whose half-lives were quantified over 5 days. The green channel was used to measure each FLAG tagged protein. The red channel was used to measure GAPDH, which was used as the loading control and method by which protein quantification was normalized. b. Western blot images demonstrating iNeurons whose protein levels were quantified at day 0 and day 5 of MG132 treatment. The green channel was used to measure either α-syn, TDP-43, or tau expression. The red channel was used to measure GAPDH, which was used as the loading control and method by which protein quantification was normalized. **Figure S7.** **Table S1.** α-Syn, TDP-43, and tau peptide library. Amino acid sequences for all peptides within the library tested in the described MSP-MS experiments in Figs. 2, 3, 4 and 5. **Table S2.** Pre-activation steps, protease concentrations, tested pHs, and timepoints for in vitro and mass spectrometry experiments. Cathepsin A, C, H, and X required pre-activation for all experiments in Figs. 1, 2, 3, 4, 5 and Fig. S1. Protease concentration and mass spectrometry timepoints were chosen and optimized based on the known rate of cleavage for each protease. **Table S3.** WT and mutant α-syn, TDP-43, and tau sequences with high rank order prediction scores of cleavage for select cathepsins. Each mutation and companion wild-type sequences for a, α-Syn; c, TDP-43, and c, tau are represented alongside the rank order prediction scores of PROSPER predicted sites of cleavage for the available cathepsins B, K, L, S, D and E. **Table S4.** Fluorogenic peptide sequences for WT and mutant α-syn, TDP-43, and tau. A, α-Syn; B, TDP-43, and C, tau amino acid sequences tested in the described fluorogenic protease activity assays in Figs. 2, 3, and 4.

**Additional file 2.**

**Additional file 3.**

## Data Availability

All data generated or analyzed during this study are included in this published article and its supplementary information files. Scripts used for data curation, analysis and visualization are available at: https://github.com/arya-shruti/Lysosomal-Protease-Project.

## Abbreviations

α-syn: α-Synuclein
TDP-43: TAR DNA-binding protein 43
PD: Parkinson's disease
ALS: Amyotrophic lateral sclerosis
FTD: Frontotemporal dementia
WT: Wild type
IPSC: Induced pluripotent stem cell
INeuron: IPSC-derived neurons
CTSA: Cathepsin A
CTSB: Cathepsin B
CTSC: Cathepsin C
CTSD: Cathepsin D
CTSE: Cathepsin E
CTSF: Cathepsin F
CTSG: Cathepsin G
CTSH: Cathepsin H
CTSK: Cathepsin K
CTSL: Cathepsin L
CTSO: Cathepsin O
CTSS: Cathepsin S
CTSV: Cathepsin V
CTSX: Cathepsin X
AEP: Asparagine endopeptidase
MSP-MS: Multiplexed substrate profiling by mass spectrometry
MS/MS: Tandem mass spectrometry
