# Correction: Mutations in α-synuclein, TDP-43 and tau prolong protein half-life through diminished degradation by lysosomal proteases

**DOI:** 10.1186/s13024-023-00629-0

**Published:** 2023-05-30

**Authors:** Paul J. Sampognaro, Shruti Arya, Giselle M. Knudsen, Emma L. Gunderson, Angelica Sandoval-Perez, Molly Hodul, Kathryn Bowles, Charles S. Craik, Matthew P. Jacobson, Aimee W. Kao

**Affiliations:** 1 Memory and Aging Center, Department of Neurology, University of California, San Francisco, CA USA; 2 Neuromuscular Division, Department of Neurology, University of California, San Francisco, CA USA; 3 Alaunus Biosciences, Inc, South San Francisco, CA USA; 4 Department of Pharmaceutical Chemistry, University of California, San Francisco, San Francisco, CA USA; 5 Department of Genetics and Genomic Sciences, Icahn School of Medicine at Mount Sinai, New York, USA; 6 UK Dementia Research Institute at the University of Edinburgh, Edinburgh Medical School, Edinburgh, UK

Molecular Neurodegeneration (2023) 18:29

10.1186/s13024-023-00621-8

The original article [1] contained a typo in author, Kathryn Bowles’ name which has since been amended.
