# Supplementary material for: Mutations in α-synuclein, TDP-43 and tau prolong protein half-life through diminished degradation by lysosomal proteases
Source: Mol Neurodegener. 2023 May 2;18:29. doi: 10.1186/s13024-023-00621-8 (PMC10155372; doi:10.1186/s13024-023-00621-8)
Supplement: Supplementary file 1 — Additional file 1: Figure S1. Proteases that do not cleave α-syn, TDP-43, or tau are active against a fluorogenic casein substrate. Fluorogenic protease activity assays using casein at pH 4.5 was performed on all enzymes that do not cleave α-syn, TDP-43, or tau. The values obtained were normalized to timepoint t = 0 and plotted relative to activity. All enzymes, including both cysteine and serine proteases, show activity against the optimized fluorogenic peptides compared to the substrate only control. Figure S2. PROSPER analysis predicts multiple cathepsin cleavage sites with α-syn, TDP-43, and tau. Cathepsins Gand K, the lysosomal proteases available in the PROSPER database, demonstrate many sites of cleavage throughout the α-syn, TDP-43, and tau substrates. Yellow highlighted amino acids represent the P1 site of predicted CTSK cleavage. Light blue highlighted amino acids represent the P1 of predicted CTSG cleavage. Figure S3. Cathepsin activity assay Vmax tables of α-syn, TDP-43, and tau wild type versus mutant substrates. a, α-Syn, b, TDP-43, and c, tau. Vmax values are listed in nM/min. An arbitrary value of 2000 value was given to protease-enzyme substrate interactions that were too fast to be modeled. Figure S4. Doxycycline-induced expression of mutant α-syn, TDP-43, and tau in differentiated SHSY-5Y cells. a, α-Syn; b, TDP-43; and c, tau. Western blot images demonstrating induced expression of each FLAG-tagged protein by our plasmid constructs with 24 h of doxycycline treatment results in measurable protein whose half-life can be readily quantified over days. These results also demonstrate minimal leakiness of these constructs in the absence of doxycycline. Figure S5. Proteasomal inhibitor, MG132, upregulates markers of autophagy. a., Western blot images demonstrating that Lamp1, cathepsin D, LCBIIb, and TFEB are all upregulated after MG132 treatment at 24, 72, and 120 h, compared to the no treatment condition. b., Quantification of the results of A. Experim [file 13024_2023_621_MOESM1_ESM.pdf]

Figure S1

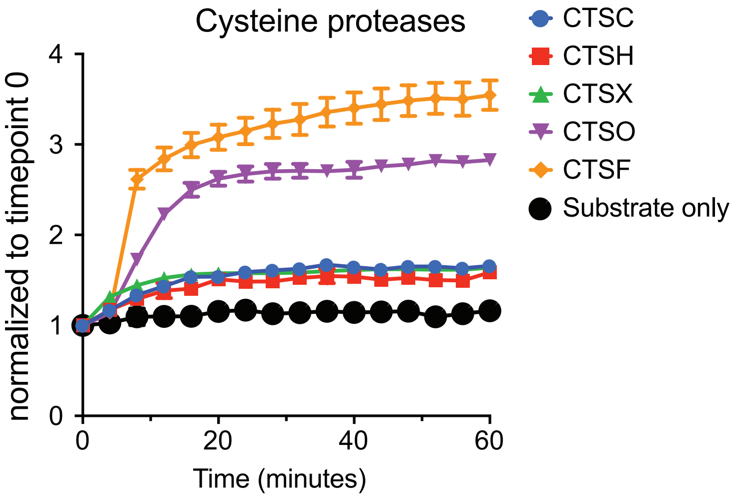
Cysteine proteases
normalized to timepoint 0
Time (minutes)
0
1
2
3
4
0
20
40
60
CTSC
CTSH
CTSX
CTSO
CTSF
Substrate only

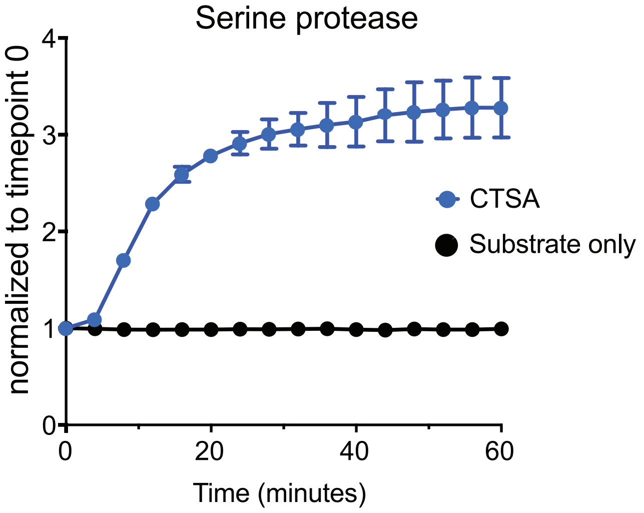
Serine protease
normalized to timepoint 0
Time (minutes)
0
1
2
3
4
0
20
40
60
CTSA
Substrate only

Figure S2

Alpha-synuclein

[1]MDVFMKGLSKAKEGVVAAAEKTKQGVAEAAGKTKEGVLYVGSKTKEGVVHGVATVAEKTKEQVTNV
GGAVVTGVTAVAQKTVEGAGSIAAATGFVKKDQLGKNEEGAPQEGILEDMPVDPDNEAYEMPSEEG
YQDYEPEA[140]

Tau

[1]MAEPRQEFEVMEDHAGTYGLGDRKDQGGYTMHQDQEGDTDAGLKESPLQTPTEDGSEEPGSETSDA
KSTPTAEDVTAPLVDEGAPGKQAAAQPHTEIPEGTTAEEAGIGDTPSLEDEAAGHVTQARMVSKSK
DGTGSDDKKAKGADGKTKIATPRGAAPPGQKGQANATRIPAKTPPAPKTPPSSGEPPKSGDRSGYS
SPGSPGTPGSRSRTPSLPTPPTREPKKVAVVRTPPKSPSSAKSRLQTAPVPMPDLKNVKSKIGSTE
NLKHQPGGGKVQIINKKLDLSNVQSKCGSKDNIKHVPGGGSVQIVYKPVDLSKVTSKCGSLGNIHH
KPGGGQVEVKSEKLDFKDRVQSKIGSLDNITHVPGGGNKKIETHKLTFRENAKAKTDHGAEIVYKS
PVVSGDTSPRHLSNVSSTGSIDMVDSPQLATLADEVSASLAKQGL[441]

TDP-43

[1]MSEYIRVTEDENDEPIEIPSEDDGTVLLSTVTAQFPGACGLRYRNPVSQCMRGVRLVEGILHAPDA
GWGNLVYVVNYPKDNKRKMDETDASSAVKVKRAVQKTSDLIVLGLPWKTTEQDLKEYFSTFGEVLM
VQVKKDLKTGHSKGFGFVRFTEYETQVKVMSQRHMIDGRWCDCKLPNSKQSQDEPLRSRKVFVGRC
TEDMTEDELREFFSQYGDVMDVFIPKPFRAFAFVTFADDQIAQSLCGEDLIIKGISVHISNAEPKH
NSNRQLERSGRFGGNPGGFGNQGGFGNSRGGGAGLGNNQGSNMGGGMNFGAFSINPAMMAAAQAAL
QSSWGMMGMLASQQNQSGPSGNNQNQGNMQREPNQAFGSGNNSYSGSNSGAAIGWGSASNAGSGSG
FNGGFGSSMDSKSSGWGM[414]

X Cleaved by CTSK after this residue (P1 position)

X Cleaved by CTSG after this residue (P1 position)

a

Asyn Vmax (nM/min)				
	WT	Mutant	WT	Mutant
	CTSD		CTSE	
A30P	0.0	0.0	0.0	0.0
E46K	0.0	0.0	0.0	0.0
G51D	0.0	0.0	0.0	0.0
A53T	0.0	0.0	45.2	17.2
	CTSB		CTSF	
A30P	149.7	116.7	0.0	0.0
E46K	172.2	176.1	0.0	0.0
G51D	152.4	144.1	0.0	0.0
A53T	149.3	175.7	0.0	0.0
	CTSL		CTSK	
A30P	130.6	184.6	59.1	109.7
E46K	30.9	45.0	2000.0	2000.0
G51D	34.0	12.2	2000.0	0.0
A53T	83.1	73.0	44.9	0.0
	CTSS		CTSV	
A30P	161.8	252.7	323.6	397.8
E46K	0.0	0.0	16.0	16.1
G51D	0.0	0.0	148.1	133.4
A53T	76.0	77.4	228.6	255.5
	AEP			
A30P	0.0	0.0		
E46K	0.0	0.0		
G51D	0.0	0.0		
A53T	0.0	0.0		

b

TDP-43 Vmax (nM/min)				
	WT	Mutant	WT	Mutant
	CTSD		CTSE	
G298S	0.0	0.0	0.0	0.0
A315T	0.0	0.0	60.0	23.4
A321G	135.2	112.5	34.1	21.0
Q331K	0.0	0.0	0.0	0.0
M337V	0.0	0.0	0.0	0.0
	CTSB		CTSF	
G298S	61.2	45.0	0.0	0.0
A315T	131.0	333.4	0.0	0.0
A321G	945.3	1116.8	0.0	0.0
Q331K	181.0	0.0	0.0	0.0
M337V	45.4	90.0	0.0	0.0
	CTSL		CTSK (1nM)	
G298S	2000.0	2000.0	87.3	63.7
A315T	2000.0	2000.0	2000.0	2000.0
A321G	2000.0	2000.0	99.2	108.3
Q331K	220.0	0.0	133.3	2000.0
M337V	2000.0	2000.0	52.5	37.4
	CTSS		CTSV	
G298S	38.2	46.8	181.0	196.3
A315T	93.8	43.5	205.0	155.4
A321G	145.3	159.1	82.2	79.0
Q331K	306.5	2000.0	216.3	26.5
M337V	115.2	61.9	161.7	124.9
	AEP			
G298S	0.0	0.0		
A315T	0.0	0.0		
A321G	0.0	0.0		
Q331K	0.0	0.0		
M337V	0.0	0.0		

c

Tau Vmax (nM/min)				
	WT	Mutant	WT	Mutant
	CTSD		CTSE	
K257T	0.0	0.0	0.0	0.0
N279K	2000.0	2000.0	2000.0	2000.0
P301S	0.0	0.0	0.0	0.0
S305N	0.0	0.0	53.5	0.0
	CTSB		CTSF	
K257T	2000.0	2000.0	40.4	0.0
N279K	2000.0	2000.0	0.0	0.0
P301S	140.4	198.8	0.0	0.0
S305N	16.3	8.6	0.0	0.0
	CTSL		CTSK (1 nM)	
K257T	2000.0	2000.0	289.7	170.0
N279K	2000.0	2000.0	72.2	120.5
P301S	2000.0	2000.0	0.0	0.0
S305N	2000.0	2000.0	0.0	0.0
	CTSS		CTSV	
K257T	23.6	58.5	0.0	0.0
N279K	20.7	0.0	0.0	0.0
P301S	0.0	0.0	65.0	45.9
S305N	23.8	28.5	126.9	146.1
	AEP			
K257T	42.8	29.1		
N279K	95.7	0.0		
P301S	0.0	0.0		
S305N	0.0	0.0		

Figure S4

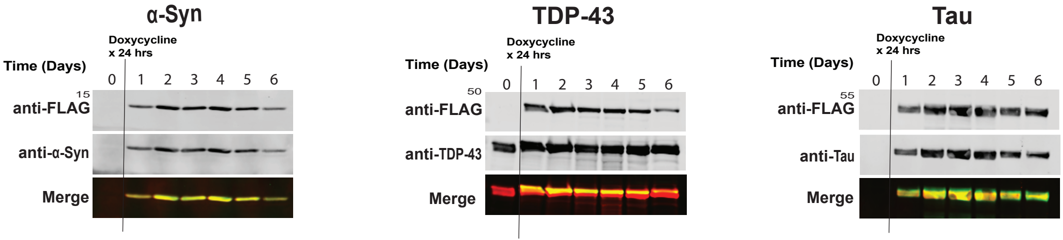
α-Syn
Doxycycline x 24 hrs
Time (Days)
0 1 2 3 4 5 6
15
anti-FLAG
anti-α-Syn
Merge
TDP-43
Doxycycline x 24 hrs
Time (Days)
0 1 2 3 4 5 6
50
anti-FLAG
anti-TDP-43
Merge
Tau
Doxycycline x 24 hrs
Time (Days)
0 1 2 3 4 5 6
55
anti-FLAG
anti-Tau
Merge

Figure S5

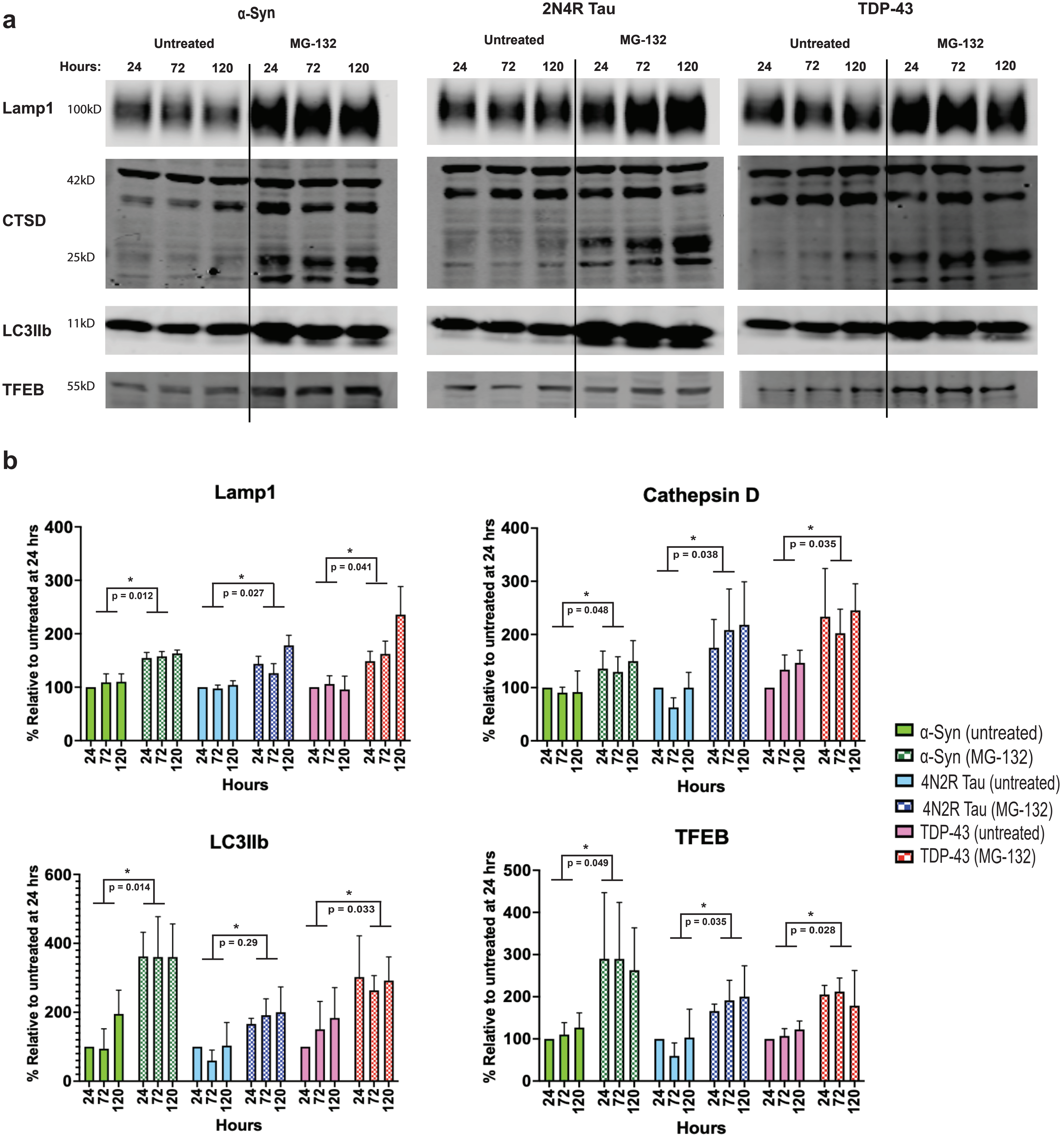
a
α-Syn
2N4R Tau
TDP-43
Untreated
MG-132
Hours:
24
72
120
Lamp1
100kD
CTSD
42kD
25kD
LC3IIb
11kD
TFEB
55kD
b
Lamp1
Cathepsin D
LC3IIb
TFEB
% Relative to untreated at 24 hrs
Hours
p = 0.012
p = 0.027
p = 0.041
p = 0.048
p = 0.038
p = 0.035
p = 0.014
p = 0.29
p = 0.033
p = 0.049
p = 0.035
p = 0.028
α-Syn (untreated)
α-Syn (MG-132)
4N2R Tau (untreated)
4N2R Tau (MG-132)
TDP-43 (untreated)
TDP-43 (MG-132)

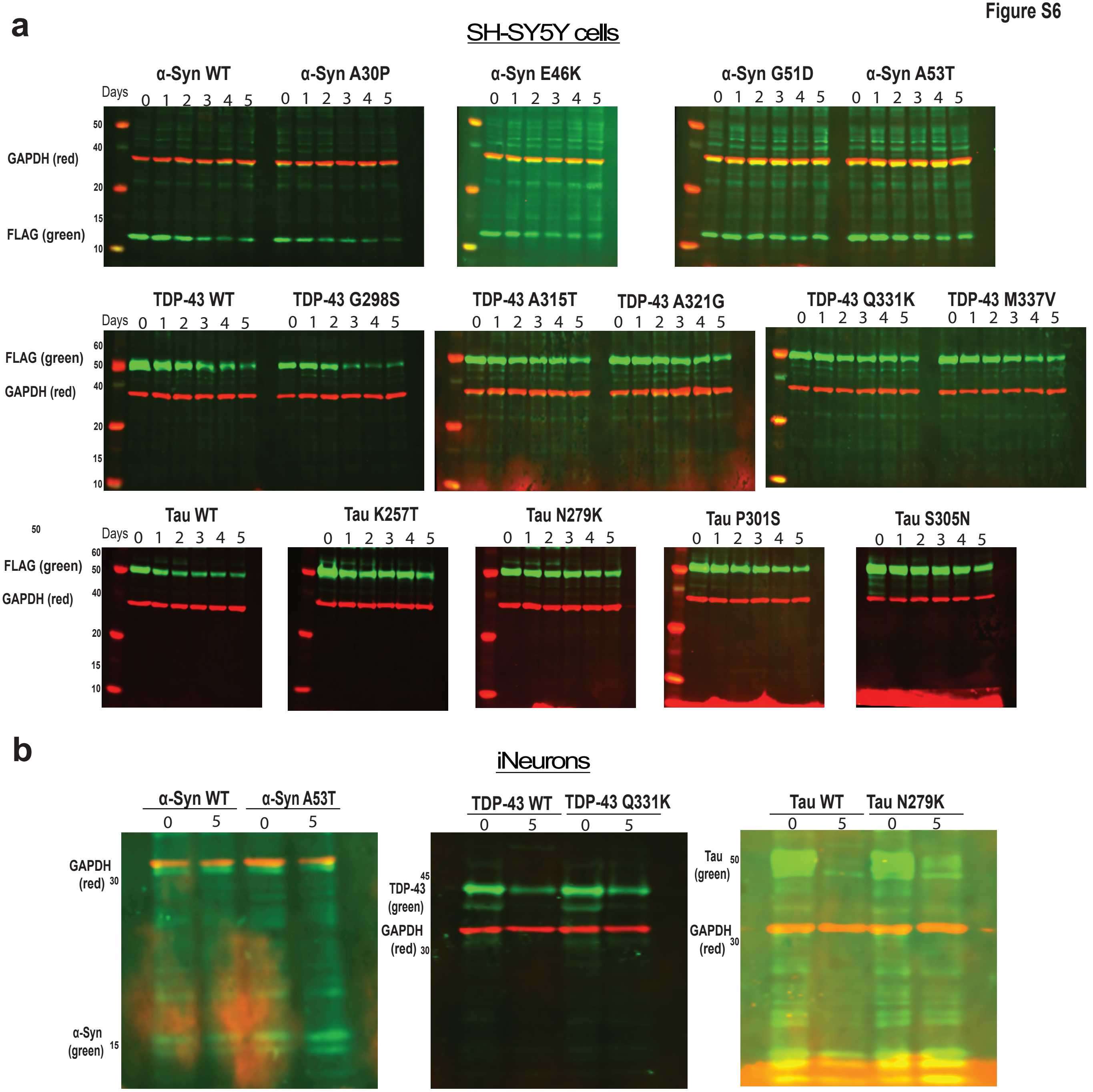
a
SH-SY5Y cells
α-Syn WT
α-Syn A30P
α-Syn E46K
α-Syn G51D
α-Syn A53T
Days
0 1 2 3 4 5
GAPDH (red)
FLAG (green)
50
40
20
15
10
TDP-43 WT
TDP-43 G298S
TDP-43 A315T
TDP-43 A321G
TDP-43 Q331K
TDP-43 M337V
60
Tau WT
Tau K257T
Tau N279K
Tau P301S
Tau S305N
b
iNeurons
α-Syn WT
α-Syn A53T
0 5 0 5
GAPDH
(red) 30
α-Syn
(green) 15
TDP-43 WT
TDP-43 Q331K
45
TDP-43
(green)
Tau WT
Tau N279K
Tau
(green) 50

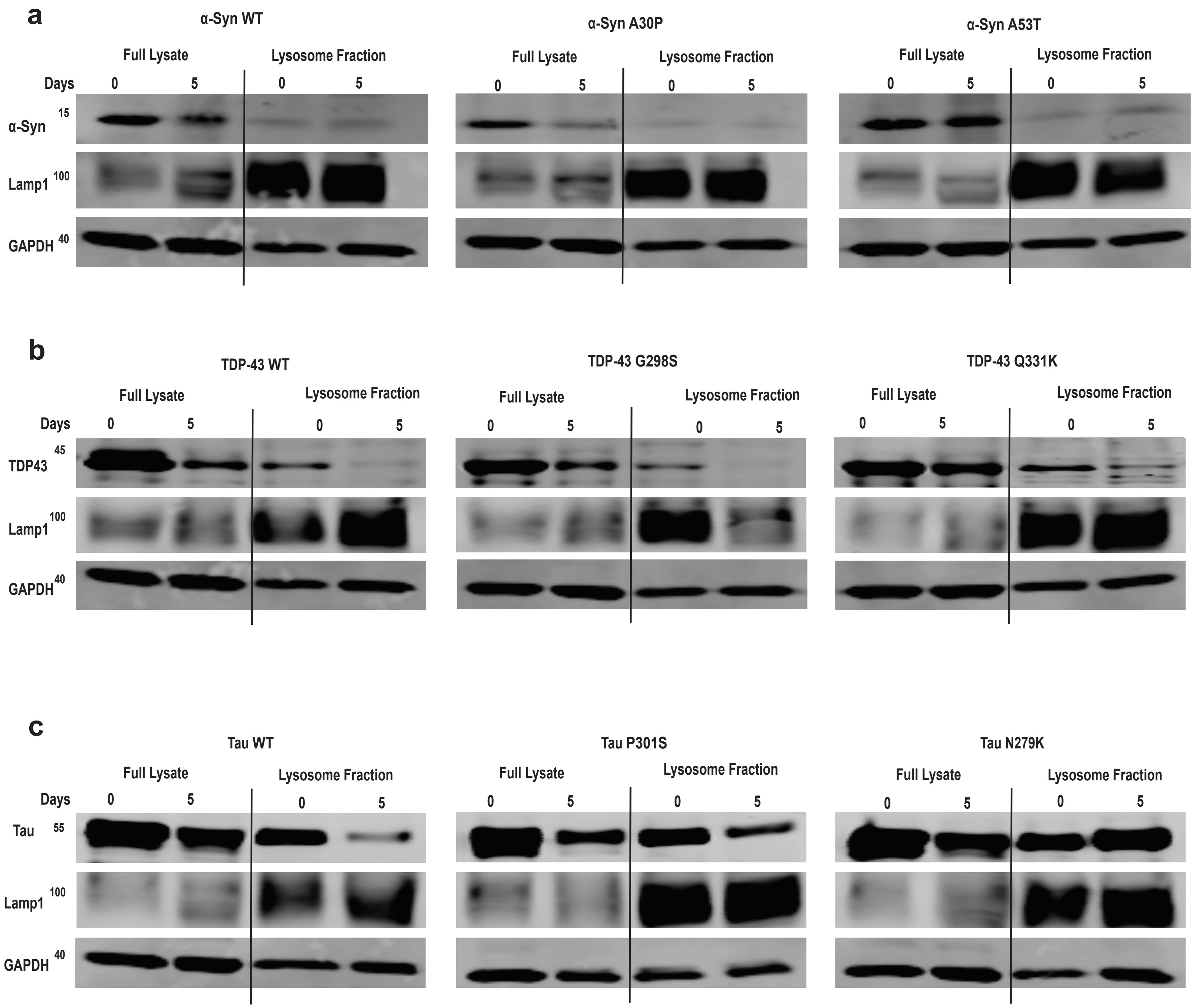
a
α-Syn WT
α-Syn A30P
α-Syn A53T
Full Lysate
Lysosome Fraction
Days
0
5
α-Syn
15
Lamp1
100
GAPDH
40
b
TDP-43 WT
TDP-43 G298S
TDP-43 Q331K
TDP43
45
c
Tau WT
Tau P301S
Tau N279K
Tau
55

Table S1

Sequence Name	peptide sequence	Sequence Name	peptide sequence	Sequence Name	peptide sequence
alpha-syn_1-18	REGMDVFMKGLSKAKEGVVAAGGR	tau_1-18	RGGMAEPRQEFEVMEDHAGTYGKR	tdp43_1-18	RKGMSEYIRVTEDENDEPIEIGKR
alpha-syn_14-31	RNKGVVAAAEKTKQGVAEAAGNGR	tau_14-31	RKGHAGTYGLGDRKDQGGYTMGSR	tdp43_14-31	RKGEPIEIPSEDDGTVLLSTVGKR
alpha-syn_27-44	RDPAEAAGKTKEGVLYVGSKTGSR	tau_27-44	RKTGGYTMHQDQEGDTDAGLKGQR	tdp43_27-44_C39A	RDGLLSTVTAQFPGAAGLRYRGDK
alpha-syn_40-57	RDGVGSKTKEGVVHGVATVAEGNR	tau_40-57	RPGDAGLKESPLQTPTEDGSEGKR	tdp43_40-57_C50A	RDGGLRYRNPVSQAMRGVRLVGDR
alpha-syn_53-70	RGGATVAEKTKEQVTNVGGAVGKR	tau_53-70	RKGEDGSEEPGSETSDAKSTPGGR	tdp43_53-70	RSGGVRLVEGILHAPDAGWGNGKK
alpha-syn_66-83	RKGVGGAVVTGVTAVAQKTVEGSR	tau_66-83	RKGAKSTPTAEDVTAPLVDEGGIR	tdp43_66-83	RDGAGWGNLVYVVNYPKDNKRGDR
alpha-syn_79-96	RGGQKTVEGAGSIAAATGFVKGSR	tau_79-96	RKGLVDEGAPGKQAAAQPHTEGKR	tdp43_79-96	RDGKDNKRKMDETDASSAVKVGSR
alpha-syn_92-109	RGGTGFVKKDQLGKNEEGAPQGNR	tau_92-109	RKGQPHTEIPEGTTAEEAGIGGQR	tdp43_92-109	RDGSAVKVKRAVQKTSDLIVLGSR
alpha-syn_105-118	RRGKGEGAPQEGILEDMPVGGKGR	tau_105-122	RKNEAGIGDTPSLEDEAAGHVGKR	tdp43_105-122	RKSDLIVLGLPWKTTEQDLKEGSR
alpha-syn_114-125	RSRKGEDMPVDPDNEAYGKGGR	tau_118-135	RDNAAGHVTQARMVSKSKDGTGGR	tdp43_118-135	RKGQDLKEYFSTFGEVLMVQVGNR
alpha-syn_121-132	RGRKGDNEAYEMPSEEGGGKGR	tau_131-148	RQGSKDGTGSDDKKAKGADGKGSR	tdp43_131-148	RSDLMVQVKKDLKTGHSKGFGGDR
alpha-syn_128-140	RNRGKPSEEGYQDYEPEAGGKR	tau_144-161	RDGGADGKTKIATPRGAAPPGGDR	tdp43_144-161	REPSKGFGFVRFTEYETQVKVGGR
		tau_157-174	RESAAPPGQKGQANATRIPAKGDR	tdp43_157-174_C173A	RDGTQVKVMSQRHMIDGRWADGGR
		tau_170-187	RDGRIPAKTPPAPKTPPSSGEGNR	tdp43_170-187_CC173AA	RSSGRWADAKLPNSKQSQDEPGKR
		tau_183-200	RGGPSSGEPPKSGDRSGYSSPGKR	tdp43_183-200_C198A	REGSQDEPLRSRKVFVGRATEGGR
		tau_196-213	RDGGYSSPGSPGTPGSRSRTPGDR	tdp43_196-213_C198A	RKSGRATEDMTEDELREFFSQGGR
		tau_209-226	REDRSRTPSLPTPPTREPKKVGDR	tdp43_209-226	RNGEFFSQYGDVMDVFIPKPFGSR
		tau_222-239	RDGEPKKVAVVRTPPKSPSSAGDR	tdp43_222-239	RKSIPKPFRAFAFVTFADDQIGSR
		tau_235-252	RDGSPSSAKSRLQTAPVPMPDGSR	tdp43_235-252_C244A	RKIADDQIAQSLAGEDLIIKGSGR
		tau_248-265	REGVPMPDLKNVKSKIGSTENGYR	tdp43_248-265	RNGLIIKGISVHISNAEPKHNGSR
		tau_261-278	RNGGSTENLKHQPGGGKVQIIGDR	tdp43_261-278	REGEPKHNSNRQLERSGRFGGTDR
		tau_274-291_C291A	RDGKVQIINKKLDLSNVQSKAGER	tdp43_274-291	RSDGRFGGNPGGFGNQGGFGNGGR
		tau_300-317	RDGVPGGGSVQIVYKPVDLSKGDR	tdp43_287-304	RTDGGFGNSRGGGAGLGNNQGGDR
		tau_313-330_C322A	RSGVDLSKVTSKAGSLGNIHHGSR	tdp43_300-317	RDGGNNQGSNMGGGMNFGAFSGGR
		tau_326-343	REGGNIHHKPGGGQVEVKSEKGNR	tdp43_313-330	RDGFGAFSINPAMMAAAQAALGNR
		tau_339-356	RGGVKSEKLDFKDRVQSKIGSGDR	tdp43_326-343	RSGAQAALQSSWGMMGMLASQGKR
		tau_352-369	REGSKIGSLDNITHVPGGGNKGTR	tdp43_339-356	RNKMLASQQNQSGPSGNNQNQGSR
		tau_365-382	RSDGGGNKKIETHKLTFRENAGSR	tdp43_352-369	RKGNNQNQGNMQREPNQAFGSGSR
		tau_378-395	RDGFRENAKAKTDHGAEIVYKGSR	tdp43_365-382	RKGQAFGSGNNSYSGSNSGAAGKR
		tau_391-408	RMGEIVYKSPVVSGDTSPRHLGSR	tdp43_378-395	RKGNSGAAIGWGSASNAGSGSGKR
		tau_404-421	RDGSPRHLSNVSSTGSIDMVDGSR	tdp43_391-408	RKGAGSGSGFNGGFGSSMDSKGNR
		tau_417-434	RKGIDMVDSPQLATLADEVSAGKK	tdp43_404-414	RNGSMDSKSSGWGMGGR
		tau_430-441	RPKDEVSASLAKQGLGSR		

Cathepsins	Pre-activation:	Protease concentrations and pHs used for mass spectrometry:	Mass spectrometry time points:
CTSA	Trans-activated with CTSL following the manufacturer's recommendation. The irreversible cysteine protease inhibitor E-64 was then added for an additional 30 min at 37 °C to allow for inhibition of CTSL.	41 nM in pH 4.5	1h, 4h
CTSB	N/A	20 nM in pH 4.5 and 5.5	1h, 4h
CTSC	Trans-activated with CTSL following the manufacturer's recommendation.The irreversible cysteine protease inhibitor E-64 was then added for an additional 30 min at 37 °C to allow for inhibition of CTSL.	N/A	1h, 4h
CTSD	N/A	10 nM in pH 3.4 and 4.5	1h, 4h
CTSE	N/A	10 nM in pH 3.4 and 4.5	1h, 4h
CTSF	N/A	50 nM in pH 4.5 buffer	2h, 6h
CTSG	N/A	N/A	1h, 4h
CSTH	Trans-activated with thermolysin following the manufacturer's recommendation. Phosphoramidon was then added to these preparations for an additional 30 min at 37 °C to allow for inhibition of thermolysin	N/A	1h, 4h
CTSK	N/A	1 nM at pH 4.5	15min, 2h
CTSL	N/A	8.0 nM at pH 4.5 and 5.5	1h, 4h
CTSO	N/A	25 nM at pH 5.5	1h, 4h
CTSS	N/A	10 nM at pH 4.5 and 5.5	1h, 4h
CTSV	N/A	20 nM at pH 3.4 and 4.5	1h, 4h
CTSX	Preincubated with dithiothreitol (DTT) following the manufacturer's recommendation.	50 nM at pH 3.4 and 4.5	1h, 4h
AEP	N/A	20 nM at pH 4.5 and 5.5	1h, 4h

α-Syn

a

<table>
<tr><th>Mutation</th><th>Peptide sequence</th><th>P1 site</th><th>CTSB</th><th>P1 site</th><th>CTSK</th><th>P1 site</th><th>CTSL</th><th>P1 site</th><th>CTSS</th><th>P1 site</th><th>CTSD</th><th>P1 site</th><th>CTSE</th></tr>
<tr><td>A53T</td><td>51-GVATV|A|E|KT-59</td><td>57</td><td>5
(-79.35)</td><td>56</td><td>2
(-31.80)</td><td>57</td><td>7
(-79.20)</td><td>57</td><td>5
(-37.13)</td><td>55</td><td>2
(-70.91)</td><td>55</td><td>9
(-83.46)</td></tr>
<tr><td>G51D</td><td>47-GV|VHGV|A|TV-55</td><td>53</td><td>19
(-100.47)</td><td>-</td><td>-</td><td>53</td><td>44
(-115.01)</td><td>53</td><td>29
(-63.75)</td><td>52</td><td>15
(-97.34)</td><td>48</td><td>20
(-101.85)</td></tr>
<tr><td>E46K</td><td>40-VG|S|KT|K|EGV-48</td><td>42</td><td>28
(-107.33)</td><td>41</td><td>28
(-71.35)</td><td>42</td><td>22
(-96.04)</td><td>44</td><td>43
(-75.58)</td><td>45</td><td>48
(-127.55)</td><td>45</td><td>48
(-137.62)</td></tr>
<tr><td>A30P</td><td>24-QG|V|A|EAAGK-32</td><td>25</td><td>37
(-115.57)</td><td>27</td><td>6
(-45.70)</td><td>27</td><td>23
(-98.26)</td><td>27</td><td>20
(-55.19)</td><td>26</td><td>21
(-106.02)</td><td>26</td><td>13
(-91.31)</td></tr>
</table>

TDP-43

b

<table>
<tr><th>Mutation</th><th>Peptide sequence</th><th>P1 site</th><th>CTSB</th><th>P1 site</th><th>CTSK</th><th>P1 site</th><th>CTSL</th><th>P1 site</th><th>CTSS</th><th>P1 site</th><th>CTSD</th><th>P1 site</th><th>CTSE</th></tr>
<tr><td>A321G</td><td>318-INPA|M|MAAA-326</td><td>321</td><td>8
(-60.10)</td><td>324</td><td>11
(-37.03)</td><td>324</td><td>8
(-56.12)</td><td>321</td><td>3
(0)</td><td>322</td><td>7
(-39.72)</td><td>322</td><td>6
(-46.79)</td></tr>
<tr><td>A315T</td><td>314-GAFSIN|PA|M-322</td><td>321</td><td>8
(-60.10)</td><td>-</td><td>-</td><td>319</td><td>30
(-74.32)</td><td>321</td><td>3
(0)</td><td>321</td><td>32
(-49.76)</td><td>321</td><td>13
(-58.00)</td></tr>
<tr><td>Q331K</td><td>327-Q|AAL|Q|SSWG-335</td><td>327</td><td>25
(-79.99)</td><td>331</td><td>2
(-10.24)</td><td>331</td><td>1
(-24.72)</td><td>331</td><td>9
(-20.45)</td><td>-</td><td>-</td><td>330</td><td>11
(-57.00)</td></tr>
<tr><td>M337V</td><td>336-MM|GM|LAS|Q|Q-344</td><td>337</td><td>26
(-80.55)</td><td>342</td><td>17
(-45.01)</td><td>-</td><td>-</td><td>343</td><td>41
(-44.74)</td><td>339</td><td>4
(-33.79)</td><td>339</td><td>8
(-49.36)</td></tr>
<tr><td>G298S</td><td>294-GGGAGLGNN-302</td><td>-</td><td>-</td><td>-</td><td>-</td><td>-</td><td>-</td><td>-</td><td>-</td><td>-</td><td>-</td><td>-</td><td>-</td></tr>
</table>

Tau

c

<table>
<tr><th>Mutation</th><th>Peptide sequence</th><th>P1 site</th><th>CTSB</th><th>P1 site</th><th>CTSK</th><th>P1 site</th><th>CTSL</th><th>P1 site</th><th>CTSS</th><th>P1 site</th><th>CTSD</th><th>P1 site</th><th>CTSE</th></tr>
<tr><td>N279K</td><td>274-KVQI|IN|KKL-282</td><td>279</td><td>8
(-73.99)</td><td>279</td><td>47
(-55.87)</td><td>279</td><td>4
(-51.57)</td><td>279</td><td>8
(-28.70)</td><td>277</td><td>5
(-65.12)</td><td>277</td><td>4
(-42.20)</td></tr>
<tr><td>S305N</td><td>304-GSVQI|V|YKP-312</td><td>308</td><td>13
(-81.71)</td><td>308</td><td>24
(-43.58)</td><td>309</td><td>10
(-65.66)</td><td>-</td><td>-</td><td>308</td><td>6
(-68.34)</td><td>308</td><td>7
(-49.00)</td></tr>
<tr><td>K257T</td><td>253-LKNVK|S|KI|G-261</td><td>258</td><td>36
(-94.04)</td><td>257</td><td>4
(-25.09)</td><td>257</td><td>6
(-62.82)</td><td>-</td><td>-</td><td>260</td><td>18
(-77.40)</td><td>260</td><td>22
(-82.14)</td></tr>
<tr><td>P301S</td><td>299-HV|PGGGSVQ-307</td><td>-</td><td>-</td><td>-</td><td>-</td><td>-</td><td>-</td><td>-</td><td>-</td><td>300</td><td>29
(-83.48)</td><td>-</td><td>-</td></tr>
</table>

Table S4

a

α-Syn	
A30P	25-QGVAEAAGK-33
	25-QGVAEPAGK-33
E46K	40-VGSKTKEGV-48
	40-VGSKTKKGV-48
G51D	47-GVVHGVATV-55
	47-GVVHDVATV-55
A53T	51-GVATVAEKT-59
	51-GVTTVAEKT-59

b

Tau	
K257T	253-LKNVKSKIG-261
	253-LKNVTSKIG-261
N279K	274-KVQIINKKL-282
	274-KVQIIKKKL-282
P301S	299-HVPGGGSVQ-307
	299-HVSGGGSVQ-307
S305N	304-GSVQIVYKP-312
	304-GNVQIVYKP-312

c

TDP-43	
G298S	294-GGGAGLGNN-302
	294-GGGASLGNN-302
A315T	314-GAFSINPAM-322
	314-GTFSINPAM-322
A321G	318-INPAMMAAA-326
	318-INPGMMAAA-326
Q331K	327-QAALQSSWG-335
	327-QAALKSSWG-335
M337V	336-MMGMLASQQ-344
	336-MVGMLASQQ-344
